# Poly-D,L-Lactic Acid Filler Increases Extracellular Matrix by Modulating Macrophages and Adipose-Derived Stem Cells in Aged Animal Skin

**DOI:** 10.3390/antiox12061204

**Published:** 2023-06-01

**Authors:** Seyeon Oh, Suk Bae Seo, Gunpoong Kim, Sosorburam Batsukh, Chul-Hyun Park, Kuk Hui Son, Kyunghee Byun

**Affiliations:** 1 Functional Cellular Networks Laboratory, Graduate School and Lee Gil Ya Cancer and Diabetes Institute, College of Medicine, Gachon University, Incheon 21999, Republic of Korea; 2 SeoAh Song Dermatologic Clinic, Seoul 05557, Republic of Korea; 3 VAIM Co., Ltd., Okcheon 29055, Republic of Korea; 4 Department of Anatomy & Cell Biology, College of Medicine, Gachon University, Incheon 21936, Republic of Korea; 5 Department of Thoracic and Cardiovascular Surgery, Gachon University Gil Medical Center, Gachon University, Incheon 21565, Republic of Korea; 6 Department of Health Sciences and Technology, Gachon Advanced Institute for Health & Sciences and Technology (GAIHST), Gachon University, Incheon 21999, Republic of Korea

**Keywords:** aged skin, NRF2, macrophage, collagen, elastic fiber

## Abstract

Poly-D,L-lactic acid (PDLLA) filler corrects soft tissue volume loss by increasing collagen synthesis in the dermis; however, the mechanism is not fully understood. Adipose-derived stem cells (ASCs) are known to attenuate the decrease in fibroblast collagen synthesis that occurs during aging, and nuclear factor (erythroid-derived 2)-like-2 factor (NRF2) increases ASCs survival by inducing M2 macrophage polarization and IL-10 expression. We evaluated the ability of PDLLA to induce collagen synthesis in fibroblasts by modulating macrophages and ASCs in a H_2_O_2_-induced cellular senescence model and aged animal skin. PDLLA increased M2 polarization and NRF2 and IL-10 expression in senescence-induced macrophages. Conditioned media from senescent macrophages treated with PDLLA (PDLLA-CM_MΦ_) reduced senescence and increased proliferation and expression of transforming growth factor-β (TGF-β) and fibroblast growth factor (FGF) 2 in senescence-induced ASCs. Conditioned media from senescent ASCs treated with PDLLA-CM_MΦ_ (PDLLA-CM_ASCs_) increased the expression of collagen 1a1 and collagen 3a1 and reduced the expression of NF-κB and MMP2/3/9 in senescence-induced fibroblasts. Injection of PDLLA in aged animal skin resulted in increased expression of NRF2, IL-10, collagen 1a1, and collagen 3a1 and increased ASCs proliferation in aged animal skin. These results suggest that PDLLA increases collagen synthesis by modulating macrophages to increase NRF2 expression, which stimulates ASCs proliferation and secretion of TGF-β and FGF2. This leads to increased collagen synthesis, which can attenuate aging-induced soft tissue volume loss.

## 1. Introduction

The main inducing factor in skin aging is oxidative stress [1]. Oxidative stress results from an imbalance between the generation and removal of reactive oxidative species (ROS). ROS accumulation leads to the degradation of biological molecules, resulting in cell death and inflammation [2]. Matrix-degrading metalloproteinases (MMPs) degrade connective tissue by destroying the extracellular matrix (ECM), resulting in reduced dermal thickness and elasticity and skin wrinkles [3]. Nuclear factor (erythroid-derived 2)-like-2 factor (NRF2) is a regulatory transcription factor that is activated by oxidative stress and plays an important role in the defense against skin damage [4]. NRF2 attenuates ECM destruction by downregulating NF-κB and reducing the expression of MMPs [5,6,7,8]. NRF2 also attenuates the inflammatory response by modulating M1/M2 macrophage polarization toward the M2 phenotype [9,10]. The expression of NRF2 or activity of NRF is reported to decrease with aging [11,12].

M1/M2 polarization in aged skin is different from that in young skin [10]. Compared with young skin, aged skin has more macrophages expressing the M1 marker CD86 and fewer macrophages expressing the M2 marker CD206 [10]. M1 macrophages secrete pro-inflammatory cytokines, including interleukin (IL)-1β and tumor necrosis factor-α (TNF-α), whereas M2 macrophages secrete anti-inflammatory cytokines such as IL-10 [13,14,15]. As an anti-inflammatory cytokine, IL-10 increases the survival and paracrine functions of various stem cells [16].

Adipose-derived stem cells (ASCs), the mesenchymal stem cells (MSCs) derived from adipose tissue, can self-renew and differentiate into other cell lineages [17]. ASCs exhibit paracrine effects by secreting various cytokines, active substances, and exosomes [18,19]. ASCs in adipose tissue can increase the migration, proliferation, and secretory activity of keratinocytes and fibroblasts involved in skin regeneration [20]. Dermal fibroblasts play an essential role in modulating ECM accumulation and remodeling [21]. Dermal fibroblasts in aged skin display reduced proliferation and collagen synthesis compared with those in young skin, and the expression of MMPs is increased in senescent dermal fibroblasts [3]. Various cytokines secreted from ASCs, including transforming growth factor (TGF)-β1, increase ECM and fibroblast collagen synthesis, which can attenuate skin wrinkling and rigidity [22]. ASCs also reduce MMP1 secretion from dermal fibroblasts [23,24].

Injectable dermal fillers are widely used for cosmetic purposes such as tissue augmentation or skin rejuvenation without surgery [25], providing a rapid volume correction that attenuates wrinkles and soft tissue defects [26]. Hyaluronic acid (HA), a natural component of soft connective tissue and dermis, is frequently used in dermal fillers [27,28,29]. Although HA has great biocompatibility, it quickly degrades in the body and has a tissue-residue time of just 6 months [30]. To avoid frequent injections, synthetic biodegradable materials that stay longer in the tissue have been developed [31,32]. Poly(lactic acid) (PLA) is a biocompatible and biodegradable material that can be used as an alternative to HA as a dermal filler [33]. PLA is classified into four types depending on the lactic acid: poly-D-lactic acid (PDLA), poly-L-lactic acid (PLLA), poly-D,L-lactic acid (PDLLA), and mesoPLA [33,34]. Among these types, PLLA and PDLLA, have been widely examined as dermal fillers [35,36,37]. In addition to their volume-occupying effect, PLA fillers promote skin rejuvenation by increasing the synthesis of collagen by fibroblasts [38,39].

We evaluated the ability of PDLLA to promote ECM synthesis and skin rejuvenation in an H_2_O_2_-induced cellular senescence model and aged animal skin. We hypothesized that PDLLA would upregulate NRF2 in dermal macrophages, leading to increased M2 polarization and IL-10 expression and reduced NF-κB and MMP expression and ECM degradation. We further expected these changes to promote ASCs proliferation and paracrine effects, resulting in increased fibroblast proliferation and ECM synthesis.

## 2. Materials and Methods

All materials used in this study were of laboratory-grade quality.

### 2.1. PDLLA Preparation

PDLLA (60 ± 10 g; VAIM Co. LTD., Okcheon, Republic of Korea) was dissolved in 1 L ethylene carbonate (EC; Sigma-Aldrich, St. Louis, MO, USA): dimethyl sulfoxide (DMSO; Sigma-Aldrich, St. Louis, MO, USA) mixture (1:9 ratio). The solution was sprayed into −10 °C or colder n-hexane (Sigma-Aldrich, St. Louis, MO, USA) to freeze the particles. The EC and DMSO remaining inside the frozen PDLLA particles were removed by dissolving them in distilled water (DW). The PDLLA particles were then filtered to obtain particles sized 10–30 μm, which were then dried. The particle size of PDLLA was determined by scanning electron microscopy (Figure S1).

The PDLLA particles were subsequently mixed with 0.6% HA solution (17:3 ratio; VAIM Co. LTD., Okcheon, Republic of Korea), and the mixture (1.3 ± 0.02 g) was placed into a 10 mL vial and lyophilized. The freeze-dried material was sterilized using ethylene oxide gas and stored [40].

### 2.2. In Vitro Model

#### 2.2.1. PDLLA Treatment of Senescence-Induced Murine Macrophages (RAW 264.7 Cells)

**Figure 1:**
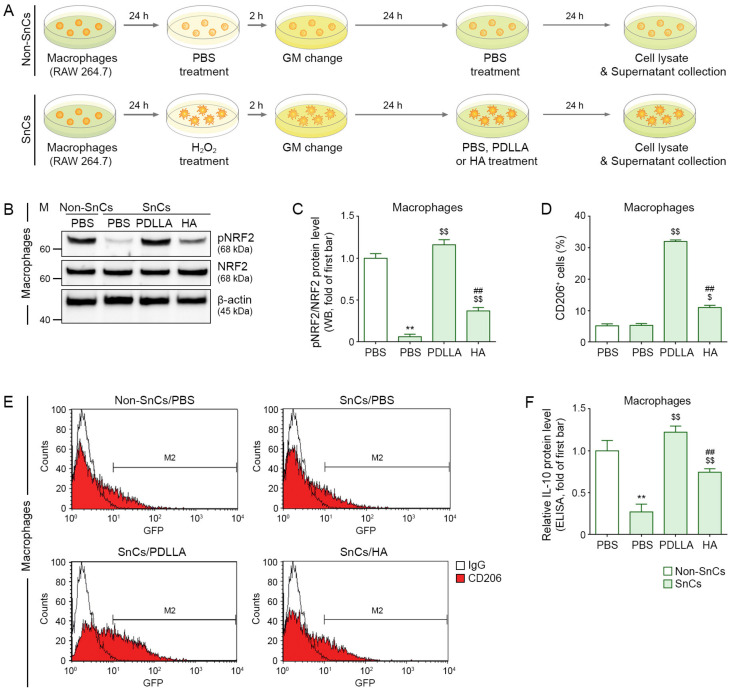
Increased pNRF2 activation, M2 polarization, and IL-10 secretion by PDLLA treatment in senescent macrophages. (**A**) This is a cell diagram to evaluate the efficacy of PDLLA or HA in senescent macrophages. After senescence was induced with H_2_O_2_ (100 µM) in macrophages (RAW 264.7) for 2 h and then cultured in GM for 24 h, the cells were treated with PDLLA or HA (200 µg/mL) for 24 h. Cell lysates and supernatants were collected and analyzed. (**B**) The protein expression of pNRF2/NRF2 was confirmed by Western blot. (**C**) This graph quantifies the data in (**B**). The expression of pNRF2/NRF2 was decreased by H_2_O_2_/PBS and increased by H_2_O_2_/PDLLA or H_2_O_2_/HA (senescent cells, SnCs) compared with that in non-senescent cells (Non-SnCs) treated with PBS/PBS. To correct for differences in protein loading, the quantification of the Western blot was normalized using β-actin as a loading control protein. For each blot, the values were expressed relative to the mean of the first bar in the graph. (**D**,**E**) The M2 polarization (CD206, M2 marker) of senescent macrophages in the presence of PDLLA or HA treatment was validated using FACS. The expression of CD206 was unchanged by H_2_O_2_/PBS and increased by H_2_O_2_/PDLLA or H_2_O_2_/HA compared with that in PBS/PBS. (**F**) The protein expression of IL-10 in the supernatant of H_2_O_2_-induced senescent macrophages was confirmed by ELISA. The expression of IL-10 was reduced by H_2_O_2_ and increased by PDLLA or HA treatment. The data were analyzed relative to the mean of the first bar of the graph. Data are presented as the mean ± SD (*n* = 3/group). **, *p* < 0.01, first bar vs. second bar; $ and $$, *p* < 0.05 and *p* < 0.01, second bar vs. third bar or fourth bar; ##, *p* < 0.01, third bar vs. fourth bar.

Murine macrophages (RAW 264.7 cells; Korea Cell Line Bank, Seoul, Republic of Korea) were routinely cultured in Dulbecco’s modified Eagle’s medium (DMEM; HyClone-Cytiva™, Marlborough, MA, USA) with 10% fetal bovine serum (FBS; Gibco™-Thermo Fisher Scientific, Rockford, IL, USA) and 1% penicillin/streptomycin (P/S; Welgene, Gyeongsan, Republic of Korea) at 37 °C in an atmosphere containing 5% CO_2_. To establish a model of macrophage senescence, a senescence induction method, which is previously used for other cells, was applied with minor modifications [41,42,43]. First, different concentrations of H_2_O_2_ (50, 100, or 200 μM) were tested to determine the optimal concentration to induce senescence (Figure S2). The three kinds of concentrations of H_2_O_2_ (50, 100, or 200 μM; Sigma-Aldrich, St. Louis, MO, USA) were treated for 2 h, washed using Dulbecco’s phosphate-buffered saline (DPBS; Gibco™-Thermo Fisher Scientific, Rockford, IL, USA), and then replaced with fresh growth medium (GM) for 24 h. Senescence was induced in murine macrophages using the H_2_O_2_ concentration (100 μM) determined from the H_2_O_2_ concentration test and then was treated with 100, 200, or 400 µg/mL of PDLLA or HA for 24 h, respectively. A total of 200 µg/mL of PDLLA or HA was determined to be the concentration that reduced senescence without causing cell death (Figure S3). RNA or protein was extracted from macrophage cell lysates, and the supernatant (Figure 1A).

#### 2.2.2. PDLLA Treatment of Senescence-Induced Human Macrophages (THP-1)

Human monocytes (THP-1; Korea Cell Line Bank, Seoul, Republic of Korea) were cultured in Roswell Park Memorial Institute (RPMI) 1640 medium (Gibco™-Thermo Fisher Scientific, Rockford, IL, USA) containing 25 mM hydroxyethyl piperazine ethane sulfonic acid (HEPES; Sigma-Aldrich, St. Louis, MO, USA), 10% FBS and 1% P/S at 37 °C with 5% CO_2_ level in a humidified incubator. Monocytes were treated with 100 ng/mL of 4β-phorbol-12-myristate-13-acetate (PMA; Sigma-Aldrich, St. Louis, MO, USA) for 24 h to differentiate into macrophages, followed by 1 μg/mL of lipopolysaccharide (LPS; Sigma-Aldrich, St. Louis, MO, USA) for 24 h to induce senescence [44]. A total of 200 µg/mL PDLLA or HA was added to the LPS-induced senescent cells for 24 h, protein was extracted from cell lysates, and the supernatant (conditioned medium from human macrophages; CM_MΦ_) was collected for co-culture with ASCs or fibroblasts (Figure S4).

#### 2.2.3. CM_MΦ_ Treatment of Senescence-Induced ASCs

Human ASCs (CEFO Bio, Seoul, Republic of Korea) were routinely cultured in CEFOgro™ human MSC growth medium (CEFO Bio, Seoul, Republic of Korea) at 37 °C in an atmosphere containing 5% CO_2_. To induce ASCs senescence, the cells were treated with 200 μM H_2_O_2_ for 2 h, washed using DPBS, and then the medium was replaced with fresh GM for 72 h [41]. During the last 24 h of the 72 h GM treatment time, a mixture of CM_MΦ_ and GM (1:1 ratio) was added. RNA or protein was isolated from cell lysates, and the supernatant (CM from ASCs; CM_ASCs_) was collected for co-culture with fibroblasts (Figure S5).

#### 2.2.4. CM_MΦ_ or CM_ASCs_ Treatment of Senescence-Induced Fibroblasts

Human fibroblasts (CCD-986Sk; American Type Culture Collection, Manassas, VA, USA) were cultured daily in Iscove’s modified Dulbecco’s medium (IMDM; Welgene, Gyeongsan, Republic of Korea), 10% FBS (Gibco™-Thermo Fisher Scientific, Rockford, IL, USA), and 1% P/S (Welgene, Gyeongsan, Republic of Korea) at 37 °C in an atmosphere containing 5% CO_2_. To induce fibroblast senescence, the cells were treated with 350 μM H_2_O_2_ for 1.5 h, washed using DPBS, and then the medium was replaced with fresh GM for 72 h [42]. During the last 24 h of the 72 h GM treatment time, a mixture of CM_MΦ_ or CM_ASCs_ and GM (1:1 ratio) was added. RNA or protein was isolated from cell lysates for further experiments (Figures S6 and S7).

### 2.3. In Vivo Model

Eight-week-old and 11-month-old male C57BL/6 mice were obtained from Orient Bio (Seongnam, Republic of Korea). Following a one-week period of acclimatization, nine-week-old mice were allocated to the young group (*n* = 3), whereas 12-month, 13-month, or 13.5-month-old mice were randomly assigned to the aging group (*n* = 3/treatment group/time point).

**Figure 2:**
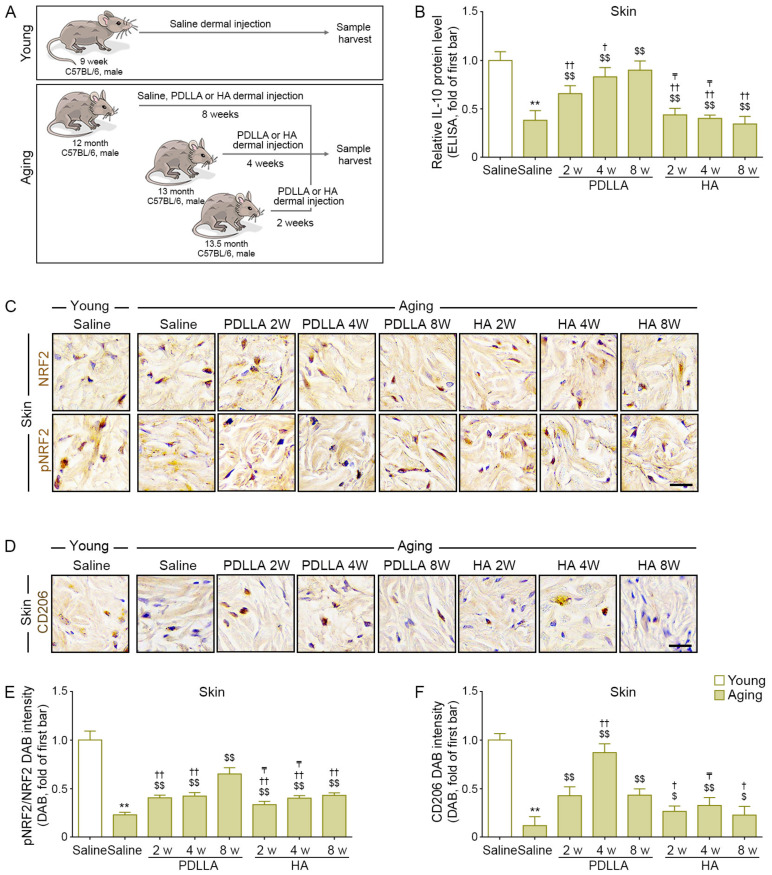
Enhanced pNRF2 activity, M2 polarization, and IL-10 secretion by PDLLA in aged skin. (**A**) This is a schematic diagram of an animal model to determine the efficacy of PDLLA or HA in aging animals. For the young group, 9-week-old male (C57BL/6) mice were injected with saline into the dermal layer at five different sites (100 μL/site). Skin tissues were collected 8 weeks later. For the aging group, 12-, 13-, and 13.5-month-old male C57BL/6 mice were injected with saline, PDLLA, or HA into the dermal layer at five different sites (100 μL/site). Skin tissues were collected 2, 4, or 8 weeks later, respectively. (**B**) The protein expression of IL-10 in the serum of aged skin was validated by ELISA. The expression of IL-10 was decreased in aged skin compared with that in young skin and was increased in aged skin by PDLLA or HA treatment. The data were represented relative to the mean of the first bar of the graph. (**C**,**D**) The expression levels of NRF2, pNRF2, and CD206 in aged skin were validated using DAB staining (scale bar = 50 μm). (**E**,**F**) This graph quantifies the data in Figure 2C,D. The expression of pNRF2/NRF2 (**E**), and CD206 (**F**) was decreased in aged skin compared with that in young skin and was increased in aged skin by PDLLA or HA treatment. The data were normalized based on the mean value of the first bar in the graph. Data are presented as the mean ± SD (*n* = 3/group). **, *p* < 0.01, first bar vs. second bar; $ and $$, *p* < 0.05 and *p* < 0.01, second bar vs. third bar~eighth bar; † and ††, *p* < 0.05 and *p* < 0.01, fifth bar vs. third bar~eighth bar; ₸, *p* < 0.05, eighth bar vs. sixth, seventh bar.

Saline, PDLLA, or HA (100 μL each) was injected into the dermis on the back of mice in the aging mice at five different sites, and skin tissues were collected 2, 4, or 8 weeks later. The animal experiment was designed to collect skin tissue from 14-month-old mice. Mice in the young group were injected with saline and harvested 8 weeks later as a control (Figure 2A).

After induction of respiratory anesthesia using 0.3% isoflurane (HANA Pharm Co., Ltd., Seoul, Republic of Korea) and 1.5% O_2_, the injected areas were shaved, and skin tissues were collected.

The mice used in this study were domesticated in an area with controlled temperature (22 ± 5 °C), and relative humidity (50 ± 10%), and a 12-h light–dark cycle. The animals had free access to a standard laboratory diet and water. This study was approved by the ethical board of the Center of Animal Care and Use and was conducted in accordance with the Institutional Animal Care and Use Committee of Gachon University (approval number: LCDI-2021-0156).

### 2.4. RNA Extraction and cDNA Synthesis

#### 2.4.1. RNA Extraction

Total RNA was extracted from the cells using the RNAiso reagent (Takara Bio Inc., Shiga, Japan) according to the manufacturer’s instructions. Briefly, cells (1 × 10^6^ cells/mL) were washed with cold PBS and lysed with RNAiso reagent. Chloroform (Samchun, Seoul, Republic of Korea) was added to the lysate, and the mixture was centrifuged at 12,000× *g* for 15 min at 4 °C to separate the aqueous and organic phases. The aqueous phase was then transferred to a fresh tube, and RNA was precipitated by adding isopropanol (Duksan, Seoul, Republic of Korea) for 10 min at room temperature (RT). The RNA was centrifugated at 12,000× *g* for 10 min at 4 °C to precipitate the pellet. Subsequently, the RNA was washed with 75% cold-ethanol (Sigma-Aldrich, St. Louis, MO, USA), and centrifuged at 7500× *g* for 5 min at 4 °C. The washed RNA pellet was air-dried until the pellet was slightly transparent and dissolved in diethyl pyrocarbonate treated water (Biosesang, Seongnam, Republic of Korea). RNA quality and quantity were determined using a NanoDrop spectrophotometer (Thermo Fisher Scientific, Rockford, IL, USA).

#### 2.4.2. cDNA Synthesis

Total RNA (1 μg) was reverse-transcribed into cDNA using the cDNA synthesis kit (Takara Bio Inc., Shiga, Japan) according to the manufacturer’s instructions. Briefly, RNA was mixed with a mixture of Oligo DT primer and dNTPs in an RNase-free DW. The reaction mixture was incubated at 65 °C for 5 min, then, the reaction mixture reacted with a mixture of reverse transcriptase and RNase inhibitor in a reaction buffer. The reaction mixture was incubated at 42 °C for 45 min, followed by 95 °C for 5 min using thermal cyclers (Bio-Rad, Hercules, CA, USA).

### 2.5. Quantitative Real-Time Polymerase Chain Reaction (qRT-PCR)

The expression levels of target genes were measured by qRT-PCR using the ROX plus SYBR green premix (Takara Bio Inc., Shiga, Japan). Gene-specific primers were designed using Primer3 software and synthesized by a commercial supplier (Cosmogenetech, Seoul, Republic of Korea) (Table S1). Briefly, cDNA samples (2.5 µL) were mixed with the SYBR green premix (5 µL), gene-specific primers (0.4 µL each for reverse and forward primer), and DW (1.7 µL) in a 384-well plate. The PCR amplification was performed using a real-time PCR instrument (Thermo Fisher Scientific, Rockford, IL, USA) with the following condition: an initial denaturation step at 95 °C for 10 min, followed by 40 cycles at 95 °C for 15 s, annealing at 60 °C for 1 min, and extension at 95 °C for 15 s. Subsequently, a melting analysis was conducted by gradually increasing the temperature from 60 °C to 95 °C at a rate of 0.075 °C per sec. Gene expression levels were analyzed using the comparative CT method (ΔΔCT) [45]. The mRNA levels were standardized to Actb, b2m, or Tbp [46] and presented relative to the levels in the first bar of each graph.

### 2.6. Cell Survival Assay

To determine the apoptotic capacity of senescent macrophages treated with PDLLA or HA, 5000 murine macrophages (RAW 264.7 cells) were seeded in each well of a 96-well culture plate (SPL Life Sciences, Pocheon, Republic of Korea). Senescence induction of murine macrophages was made as mentioned in Section 2.2.1., and senescence-induced murine macrophages were treated with PDLLA and HA by concentration (100, 200, or 400 µg/mL) for 24 h. The cell counting kit (TransGen Biotech Co., Ltd., Beijing, China) mixed with serum-free DMEM (1:9, *v*/*v*; HyClone-Cytiva™, Marlborough, MA, USA) was added, and the mixture was incubated for 4 h at 37 °C in an atmosphere containing 5% CO_2_. Optical density was measured at 450 nm using a plate reader (Multiskan SkyHigh Photometer; Thermo Fisher Scientific, Rockford, IL, USA).

### 2.7. Proliferation Assay

To confirm changes in the proliferative ability of senescent fibroblasts treated with PDLLA or HA, 2500 fibroblasts were seeded in each well of a 96-well culture plate (SPL Life Sciences, Pocheon, Republic of Korea), and the in vitro model was manufactured as described in Section 2.2.4. Cell counting kit (TransGen Biotech Co., Ltd., Beijing, China) was mixed with serum-free DMEM (1:9, *v*/*v*; Welgene, Gyeongsan, Republic of Korea), and the mixture was incubated for 4 h at 37 °C in an atmosphere containing 5% CO_2_. Optical density was measured at 450 nm using a plate reader (Multiskan SkyHigh Photometer; Thermo Fisher Scientific, Rockford, IL, USA).

### 2.8. Protein Extraction

The cells were homogenized using a glass homogenizer (Jeung do Bio & PLANT Co., Ltd., Seoul, Republic of Korea) in prepared RIPA lysis buffer supplemented with protease and phosphatase inhibitor (EzRIPA buffer kit; ATTO Corporation, Tokyo, Japan). Moreover, the frozen skin tissues were homogenized using a bead homogenizer (Allsheng Instrument, Hangzhou, China) at 6.0 m/s for 5 cycles (running for 40 s and interrupting for 45 s) in a prepared buffer. Then, the homogenates were incubated on ice for 10 min to facilitate cell lysis and protein solubilization. The samples were sonicated and centrifuged at 14,000× *g* for 15 min at 4 °C to separate the soluble protein fraction from the insoluble debris and organelles. The concentration of protein samples was measured using a bicinchoninic acid assay kit (Thermo Fisher Scientific, Rockford, IL, USA) following the manufacturer’s instructions.

### 2.9. Western Blot

For Western blot, the extracted cell lysates and skin tissues proteins (30 µg) were mixed with a sample buffer (4× LDS buffer; Thermo Fisher Scientific, Rockford, IL, USA) and sample reducing agent (Thermo Fisher Scientific, Rockford, IL, USA) and then heated at 70 °C for 10 min to denature the proteins. The denatured protein was separated using 8, 10, or 12% sodium dodecyl sulfate-polyacrylamide gel electrophoresis by MOPS buffer (Invitrogen, Waltham, MA, USA) and protein electrophoresis equipment (Bio-Rad, Hercules, CA, USA) at 200 V for 20 min and then transferred to polyvinylidene fluoride (PVDF) membranes (Merck Millipore, Burlington, MA, USA) by semi-dry transfer system (ATTO Corporation, Tokyo, Japan) at 1 A for 10 min. To prevent non-specific binding, the PVDF membranes were incubated with 5% skim milk (LPS solution, Daejeon, Republic of Korea) at RT for 1 h. After washing with tris-buffered saline with 0.1% tween 20 (TTBS), the membranes were then incubated with appropriately diluted primary antibodies (Table S2) at 4 °C overnight. The membranes were then washed with TTBS three times and incubated with horseradish peroxidase-conjugated secondary antibodies (Vector Laboratories, Burlingame, CA, USA) at RT for 2 h. The blotting membrane was developed using enhanced chemiluminescence solution (Cytiva™, Marlborough, MA, USA) on a ChemiDoc Imaging Systems (Bio-Rad, Hercules, CA, USA). All protein bands were quantified using ImageJ software (National Institutes of Health, NIH, Maryland, MD, USA). The data obtained were normalized to the β-actin (Cell signaling, Danvers, MA, USA) and expressed as fold change relative to the first bar in each graph.

### 2.10. Enzyme-Linked Immunosorbent Assay (ELISA)

The coating solution mixture (0.6% sodium bicarbonate and 0.3% sodium carbonate in DW; Sigma-Aldrich, St. Louis, MO, USA) was incubated on 96-well plates overnight at 4 °C. To prevent non-specific protein binding, 5% skim milk was incubated on the plates at RT for 4 h. The plates were then washed with PBS containing 0.1% tween 20 (TPBS). The supernatant of murine macrophages and skin tissue protein (80 µg) was added and incubated at 4 °C overnight. Unbound proteins were removed by washing with TPBS, and the plates were incubated with IL-10 primary antibodies (FineTest, Wuhan, China) at 4 °C overnight. Moreover, the cell lysates of ASCs or fibroblasts, or skin tissue protein (80 µg), were loaded and incubated at 4 °C overnight. After washing with TPBS, the plates were incubated with primary antibodies at 4 °C overnight (Table S2). After rinsing with TPBS, the plates were incubated with HRP-conjugated antibodies (Vector Laboratories, Burlingame, CA, USA) at RT for 2 h. After washing out unbound HRP-conjugated antibodies, the samples were incubated with 3,3′,5,5′-tetramethylbenzidine (TMB; Sigma-Aldrich, St. Louis, MO, USA) for 15 min for color development. The reaction was stopped by adding an equal volume of 2 M H_2_SO_4_ to the TMB solution in each well. Absorbance was measured at 450 nm using an ELISA plate reader (Multiskan SkyHigh Photometer; Thermo Fisher Scientific, Rockford, IL, USA).

### 2.11. Fluorescence-Activated Cell Sorting (FACS)

After the macrophage cell models were established as described in Section 2.2.1., the cells were harvested using trypsin-EDTA (Gibco™-Thermo Fisher Scientific, Rockford, IL, USA) and 1% penicillin/streptomycin (P/S; Welgene, Gyeongsan, Republic of Korea) and washed with phosphate-buffered saline (PBS) containing 1% FBS (FACS buffer). The cells were then resuspended in FACS buffer at a density of 1 × 10^5^ cells/mL. The macrophages were then incubated with CD206 antibody (1 μg/1 × 10^5^ cells; Santa Cruz Biotechnology, Dallas, TX, USA) and Alexa 488 antibody (5 μg/1 × 10^5^ cells; Invitrogen, Waltham, MA, USA) in FACS buffer for 30 min on ice in the dark. Unbound antibodies were removed by washing twice with FACS buffer. The stained cells were analyzed using a flow cytometer (BD FACS Calibur; BD Biosciences, Franklin Lakes, NJ, USA) equipped with appropriate filters to detect the fluorescent signals. Data were acquired and analyzed using CellQuest Pro software (BD Biosciences, Franklin Lakes, NJ, USA). The data obtained from the flow cytometry analysis were expressed as the mean fluorescence intensity and the percentage of positive cells.

### 2.12. Immunocytochemistry

Cells (3 × 10^4^ cells/well) were seeded on 8-well Lab-Tek II chamber slides (Nunc™, St. Louis, MO, USA), washed with PBS, fixed at RT for 15 min with 4% paraformaldehyde (PFA; Sigma-Aldrich, St. Louis, MO, USA), and washed again with PBS. After a 1 h blocking step to prevent non-specific binding, the cells were incubated with primary antibodies (Table S2) at 4 °C overnight. The cells were then rinsed with PBS and incubated with secondary antibodies (Invitrogen, Waltham, MA, USA) at RT for 1 h in the dark. The secondary antibodies were then washed with PBS, and the nuclei were stained with 1 µg/mL of 4′,6-diamidino-2-phenylindole (DAPI; Sigma-Aldrich, St. Louis, MO, USA) for 30 s. The coverslips were mounted using Vectashield mounting solution (Vector Laboratories, Burlingame, CA, USA), and the fluorescence was examined under a confocal microscope (Carl Zeiss 700; Carl Zeiss, Jena, Germany). For the quantification of NRF2 and CD206, the fluorescence intensity of obtained images was analyzed using Zen software (Carl Zeiss, Jena, Germany). The thresholding algorithm was applied to individual channels for analysis to identify regions of interest (green region). Thresholds were manually adjusted to ensure that only relevant structures were included in the analysis. Moreover, colocalization analysis of NF-kB was performed to assess the degree of overlap between the different fluorophores using Zen software (Carl Zeiss, Jena, Germany).

### 2.13. Paraffin-Embedded Block Preparation and Sectioning

Skin tissue samples were fixed in cold 4% PFA (Sigma-Aldrich, St. Louis, MO, USA) for 48 h. The fixed tissues were then dehydrated in increasing concentrations of ethanol, cleared in xylene, and embedded in paraffin using a tissue processor (Sakura Seiki Co., Ltd., Tokyo, Japan). Paraffin-embedded tissue blocks were sectioned into 7-μm-thick slices using a microtome (Thermo Fisher Scientific, Rockford, IL, USA), air-dried, and baked in a 60 °C oven for overnight to enhance tissue adhesion.

### 2.14. Staining with 3,3-Diaminobenzidine (DAB)

Skin tissue sections were subjected to deparaffinization and rehydration by sequentially immersing them in a series of xylene (Duksan, Seoul, Republic of Korea) and a gradient of 70–100% alcohols (Duksan, Seoul, Republic of Korea). The deparaffinized and rehydrated skin tissue sections were treated with sodium citrate buffer (pH 6; Sigma-Aldrich, St. Louis, MO, USA) for antigen retrieval. After washing with PBS, the sections were blocked with normal serum (Vector Laboratories, Burlingame, CA, USA) for 1 h at RT to prevent non-specific signals. The sections were then incubated with primary antibodies (Table S2) at 4 °C overnight and stained with the corresponding biotinylated-conjugated secondary antibodies (Vector Laboratories, Burlingame, CA, USA) at RT for 1 h and the slides were incubated with ABC reagent (Vector Laboratories, Burlingame, CA, USA) at RT for 30 min. To counterstain the nuclei, the stained slides were immersed in hematoxylin (Korea pathology technical center, Cheong Ju, Republic of Korea) for 1 min. DPX mount solution (Sigma-Aldrich, St. Louis, MO, USA) was used for mounting. All stained slides were observed under an optical microscope (Olympus, Tokyo, Japan) and scanned and captured using a slide scanner (Motic, Vancouver, Canada). All images were analyzed for DAB staining intensity using ImageJ software (NIH, Maryland, MD, USA).

### 2.15. Immunofluorescence

Antigen-retrieval-treated skin tissue slides were incubated at RT for 1 h with normal serum (Vector Laboratories, Burlingame, CA, USA) to prevent non-specific binding. The tissue sections were then incubated with primary antibodies (Table S2), rinsed with PBS, and incubated with fluorescence-conjugated secondary antibodies (Invitrogen, Waltham, MA, USA). All antibodies were incubated for 1 h in the dark. Nuclei were counterstained with DAPI (1 µg/mL; Sigma-Aldrich, St. Louis, MO, USA) at RT for 30 s. After washing with PBS, sections were mounted and covered on glass slides using a Vectashield mounting medium (Vector Laboratories, Burlingame, CA, USA). Slides were analyzed using a confocal microscope (Carl Zeiss 710; Carl Zeiss, Jena, Germany), and the obtained images were analyzed using Zen software (Carl Zeiss, Jena, Germany). To assess the degree of overlap between different fluorophores, colocalization analyses were performed.

### 2.16. Histological Analysis

#### 2.16.1. Masson’s Trichrome Staining

For collagen fiber staining, after deparaffinization as in Section 2.14., skin tissues were incubated in a Bouin solution (Scytek Laboratories, West Logan, UT, USA) at 60 °C for 1 h and rinsed with DW. The sections were then sequentially placed in a working weight solution of iron hematoxylin (Korea pathology technical center, Cheong Ju, Republic of Korea) for 5 min, Biebrich scarlet acid fuchsin solution (Scytek Laboratories, West Logan, UT, USA) for 5 min, phosphomolybdic-phosphotungstic acid solution (Scytek Laboratories, West Logan, UT, USA) for 12 min, and aniline blue solution (Scytek Laboratories, West Logan, UT, USA) for 3 min at RT. The stained slides were mounted with DPX mount solution (Sigma-Aldrich, St. Louis, MO, USA) and observed under an optical microscope (Olympus, Tokyo, Japan) equipped with a slide scanner (Motic, Vancouver, Canada). All images were analyzed for collagen fiber density using ImageJ software (NIH, Maryland, MD, USA).

#### 2.16.2. Verhoeff Staining

For elastic fiber staining, deparaffinized skin tissues were loaded with an elastic staining solution (Scytek Laboratories, West Logan, UT, USA) at RT for 15 min and then rinsed with tap water. The slides were then incubated with 20 drops of 2% ferric chloride differentiation solution (Scytek Laboratories, West Logan, UT, USA), washed with DW, and mounted using DPX mount solution (Sigma-Aldrich, St. Louis, MO, USA). The mounted slides were observed under an optical microscope equipped with a slide scanner (Motic, Vancouver, Canada) to confirm positive staining. Captured images were analyzed for elastin fiber densities using ImageJ software (NIH, Maryland, MD, USA).

#### 2.16.3. Herovici Staining

Mature collagen, newly generated collagen, and reticulin were identified in skin tissues using a Herovici Collagen Stain Kit (Scytek Laboratories, West Logan, UT, USA). Deparaffinized slides were incubated in Weigert’s iron hematoxylin for 8 min to stain the nuclei and then washed with tap water and DW. The slides were then treated with Herovici solution for 2 min and mounted with DPX mounting solution (Sigma-Aldrich, St. Louis, MO, USA). Slides were observed under a microscope and imaged using a slide scanner (Motic, Vancouver, Canada). Newly synthesized collagen was stained blue, whereas mature collagen was stained red. The staining densities were analyzed using ImageJ software (NIH, Maryland, MD, USA).

### 2.17. Statistical Analysis

Statistical significance was identified by the Kruskal–Wallis test for comparisons of each group, followed by a post hoc Mann–Whitney U test using SPSS software (version 22; IBM Corporation, Armonk, NY, USA). All results are presented as the mean ± standard deviation, and all experiments were repeated in triplicate. Statistical significance was indicated as follows, with *p* < 0.05 for one symbol, *p* < 0.01 for two symbols, and *p* < 0.001 for three symbols in the figure legends.

*: first bar vs. second bar

$: second bar vs. third bar~eighth bar

#: third bar vs. fourth bar

†: fifth bar vs. third bar~eighth bar

₸: eighth bars vs. sixth or seventh bar

## 3. Results

### 3.1. PDLLA Reduced Macrophage Senescence

We first evaluated the ability of PDLLA to reduce macrophage senescence using the H_2_O_2_-induced model of cellular senescence, which is the most widely used in vitro aging model available [41,42,43]. RAW 264.7 cells (a murine monocyte/macrophage cell line) were exposed to H_2_O_2_ for 2 h and then treated with PBS, HA, or PDLLA (Figure 1A). Cellular senescence was evaluated by measuring p21 and p16 expression [43]. The H_2_O_2_ treatment increased p21 and p16 expression in the macrophages compared with the PBS treatment alone, indicating that the H_2_O_2_ treatment-induced senescence. Senescent macrophages treated with PDLLA or HA had lower p21 and p16 expression than senescent macrophages treated with PBS, and the reduction in p21 and p16 was greater in the macrophages treated with PDLLA (Figure S8).

### 3.2. PDLLA Upregulated NRF2, CD206, and IL-10 in Senescent Macrophages

NRF2 expression in macrophages was evaluated by Western blot and ICC staining. H_2_O_2_ treatment resulted in a lower ratio of phosphorylated NRF2 to NRF2 (pNRF2/NRF2) compared with PBS treatment alone. The pNRF2/NRF2 ratio was higher in senescent macrophages (RAW 264.7 cells) treated with PDLLA than in senescent macrophages treated with HA or PBS (Figure 1B,C and Figure S9A,B).

M2-type macrophages were evaluated by FACS and ICC staining of CD206. H_2_O_2_ treatment did not cause a significant change in the number of CD206-positive cells compared with PBS treatment alone. PDLLA or HA treatment of senescent macrophages resulted in increased numbers of CD206-positive cells compared with PBS treatment of senescent macrophages, and the increase was greater after PDLLA treatment than after HA treatment (Figure 1D,E and Figure S9A,C).

IL-10 levels were evaluated by ELISA of supernatants from macrophage cultures. H_2_O_2_ treatment resulted in lower IL-10 levels than PBS treatment alone. PDLLA or HA treatment of senescent macrophages resulted in higher IL-10 levels than PBS treatment of senescent macrophages, and the increase was greater in the PDLLA-treated macrophages than in the HA-treated macrophages (Figure 1F).

### 3.3. PDLLA Upregulated NRF2, CD206, and IL-10 in Aged Skin

We determined the effect of PDLLA on aged animal skin depending on the time after injection. Twelve-month-old mice were injected with saline, PDLLA, or HA, and the skin was harvested 8 weeks after injection. Thirteen-month-old mice were injected with PDLLA or HA, and the skin was harvested 4 weeks after injection. Thirteen-and-a-half-month-old mice were injected with PDLLA or HA, and the skin was harvested 2 weeks after injection. Young (9-week-old) mice were injected with saline, and the skin was harvested 8 weeks after injection (Figure 2A). Because the main purpose was to determine if PDLLA affects aged skin by increasing NRF2 expression, which was reported to decline during aging, we did not inject the young mice with PDLLA or HA.

IL-10 levels in saline-injected aged skin were lower than those in saline-injected young skin at 8 weeks after injection. The IL-10 levels in saline-injected aged skin at 8 weeks after injection were lower than those in PDLLA- or HA-injected aged skin at 2, 4, or 8 weeks after injection. At the same time points after injection, the IL-10 levels in PDLLA-injected skin were higher than those in HA-injected skin. The IL-10 levels increased over time in PDLLA-injected skin but not in HA-injected skin (Figure 2B).

The pNRF2/NRF2 ratio was lower in aged skin than in young skin at 8 weeks after saline injection. The pNRF2/NRF2 ratio in saline-injected aged skin at 8 weeks after injection was lower than that in PDLLA- or HA-injected aged skin at 2, 4, or 8 weeks after injection. At the same time points after injection, the pNRF2/NRF2 ratio was higher in PDLLA-injected aged skin than in HA-injected aged skin. The pNRF2/NRF2 ratio increased over time in both PDLLA-injected skin and HA-injected skin (Figure 2C,E).

CD206 levels in saline-injected aged skin were lower than those in saline-injected young skin at 8 weeks after injection. The CD206 levels in saline-injected aged skin at 8 weeks after injection were lower than those in PDLLA- or HA-injected aged skin at 2, 4, or 8 weeks after injection. CD206 expression was highest at 4 weeks after injection in both PDLLA-injected skin and HA-injected skin (Figure 2D,F).

### 3.4. PDLLA Reduced ASCs Senescence by Modulating Macrophages

We used an in vitro model to evaluate the ability of PDLLA to attenuate ASCs senescence by modulating macrophages. Since human ASC was used, further experiments which evaluated the function of macrophage on ASCs were performed with human monocyte (THP-1 cells) to avoid immunological effects caused by differences between species. We treated LPS to THP-1 for inducing cellular senescence (Figure S4) [44]. LPS treatment resulted in increased p21 and p16 in the THP-1. Expression of p21 and p16 was decreased by treating PDLLA or HA (Figure S10).

We obtained CM from macrophages (THP-1 cells; CM_MΦ_) that were treated with PBS (PBS/PBS-CM_MΦ_). We then used the PBS/PBS-CM_MΦ_ to treat ASCs that were previously applied with PBS (control ASCs group). We also obtained CM from macrophages that were treated first with LPS, and then with either PBS, PDLLA, or HA (LPS/PBS-CM_MΦ_, LPS/PDLLA-CM_MΦ_, and LPS/HA-CM_MΦ_, respectively). We then used those CM to treat ASCs that were previously applied with H_2_O_2_ (Figure S5). Because we treated the ASCs with a mixture of CM_MΦ_ and GM, we verified that the culture mixture itself did not change the character of the ASCs. Expression of CD166, an ASCs marker, was not changed by the use of CM_MΦ_ and GM compared to GM alone (Figure S11).

The expression of p21 and p16 in the control ASCs was lower than that in each of the H_2_O_2_-treated ASCs groups. Among the H_2_O_2_-treated ASCs groups, the p21 and p16 expression in the group treated with LPS/PDLLA-CM_MΦ_ was lower than that in the groups treated with LPS/PBS-CM_MΦ_ or LPS/HA-CM_MΦ_ (Figure S12). These results suggested that PDLLA reduced ASCs senescence by modulating macrophages.

### 3.5. PDLLA Increased ASCs Proliferation and Paracrine Effects on Dermal Fibroblasts

**Figure 3:**
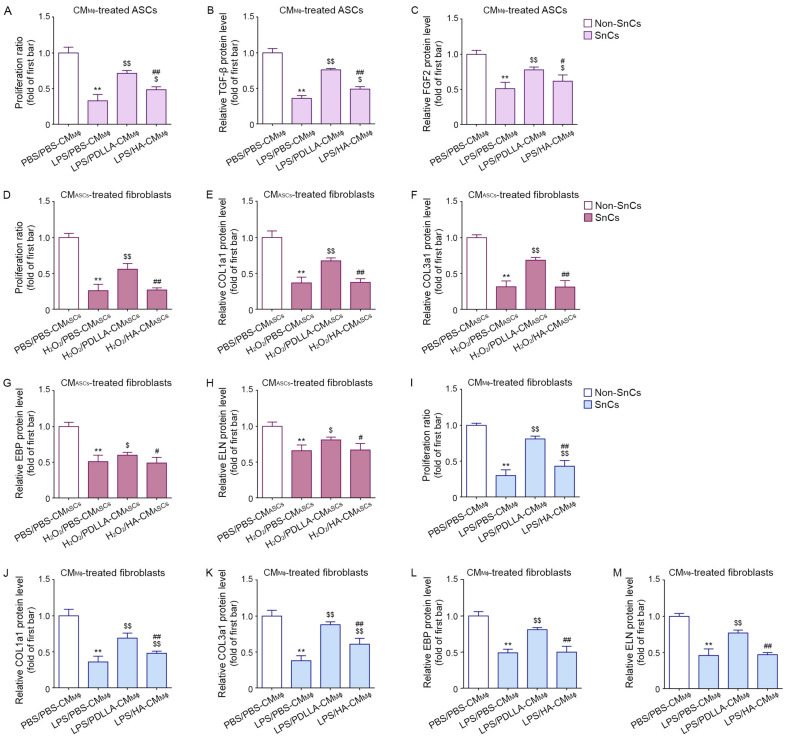
The modulating effect of PDLLA on proliferation and collagen synthesis of senescent fibroblasts affected with ASCs and macrophage. (**A**) To evaluate the ability of PDLLA to increase ASCs proliferation by modulating macrophages, proliferation assays were performed in senescent ASCs. The proliferation ratio was reduced by LPS/PBS-CM_MΦ_ and increased by LPS/PDLLA-CM_MΦ_ or LPS/HA-CM_MΦ_ (senescent cells, SnCs) compared with that in non-senescent ASCs (Non-SnCs) treated with conditioned media from non-senescent macrophages (PBS/PBS-CM_MΦ_). (**B**,**C**) The protein expression of TGF-β (**B**) and FGF2 (**C**) in ASCs was analyzed by ELISA. The expression was reduced by LPS/PBS-CM_MΦ_ and increased by LPS/PDLLA-CM_MΦ_ or LPS/HA-CM_MΦ_. (**D**) To evaluate the ability of PDLLA to increase fibroblast proliferation by modulating macrophages and ASCs, proliferation assays were performed in senescent fibroblasts. The proliferation ratio of fibroblasts was reduced by H_2_O_2_/PBS-CM_ASCs_ and increased by H_2_O_2_/PDLLA-CM_ASCs_ or H_2_O_2_/HA-CM_ASCs_ in SnCs. (**E**–**H**) The protein expression of COL1a1 (**E**), COL3a1 (**F**), EBP (**G**), and ELN (**H**) was analyzed by ELISA. The expression was reduced by H_2_O_2_/PBS-CM_ASCs_ and increased by H_2_O_2_/PDLLA-CM_ASCs_ or H_2_O_2_/HA-CM_ASCs_ in SnCs. (**I**) To evaluate the ability of PDLLA to increase fibroblast proliferation by modulating macrophages, proliferation assays were performed in senescent fibroblasts. The proliferation ratio of fibroblasts was reduced by LPS/H_2_O_2_-CM_ASCs_ and increased by LPS/PDLLA-CM_ASCs_ or LPS/HA-CM_ASCs_ in SnCs. (**J**–**M**) The protein expression of COL1a1 (**J**), COL3a1 (**K**), EBP (**L**), and ELN (**M**) was analyzed by ELISA. The expression was reduced by LPS/H_2_O_2_-CM_ASCs_ and increased by LPS/PDLLA-CM_ASCs_ or LPS/HA-CM_ASCs_ in SnCs. The data were represented relative to the mean of the first bar of the graph. Data are presented as the mean ± SD (*n* = 3/group). **, *p* < 0.01, first bar vs. second bar; $ or $$, *p* < 0.05 or *p* < 0.01, second bar vs. third bar or fourth bar; # or ##, *p* < 0.05 or *p* < 0.01, third bar vs. fourth bar.

Next, we evaluated the ability of PDLLA to increase ASCs proliferation by modulating macrophages. The proliferation ratio of the control ASCs was higher than that of each of the H_2_O_2_-treated ASCs groups. Among the H_2_O_2_-treated ASCs groups, the proliferation ratio of the group treated with LPS/PDLLA-CM_MΦ_ was higher than those of the groups treated with LPS/PBS-CM_MΦ_ or LPS/HA-CM_MΦ_ (Figure 3A).

Paracrine factors from ASCs, such as TGF-β and fibroblast growth factor (FGF)2, can stimulate fibroblast proliferation [47,48,49,50]. The expression of TGF-β and FGF2 was higher in the control ASCs than in any of the H_2_O_2_-treated ASCs groups. Among the H_2_O_2_-treated ASCs groups, the group treated with LPS/PDLLA-CM_MΦ_ had higher TGF-β and FGF2 expression than those treated with LPS/PBS-CM_MΦ_ or LPS/HA-CM_MΦ_ (Figure 3B,C). These results suggested that macrophages modulated by PDLLA attenuated the reduction of proliferation ability and paracrine function in senescent ASCs.

### 3.6. PDLLA Reduced Fibroblast Senescence by Modulating ASCs

Next, we evaluated whether the modulation of ASCs function by CM from PDLLA-treated macrophages (THP-1 cells) could affect fibroblast senescence in an in vitro model. As a control, we cultured PBS-treated fibroblasts with CM from PBS-treated ASCs cultured with PBS/PBS-CM_MΦ_ (PBS/PBS-CM_ASCs_). In addition, we cultured H_2_O_2_-treated fibroblasts with CM from H_2_O_2_-treated ASCs cultured with LPS/PBS-CM_MΦ_, LPS/PDLLA-CM_MΦ_, or LPS/HA-CM_MΦ_ (H_2_O_2_/PBS-CM_ASCs_, H_2_O_2_/HA-CM_ASCs_, and H_2_O_2_/PDLLA-CM_ASCs_, respectively) (Figure S6). Because we cultured the fibroblasts in a mixture of CM_ASCs_ and growth medium, we confirmed that the mixture did not change the expression of vimentin, a fibroblast marker, compared to the growth medium alone (Figure S13). We then measured the senescence markers p21 and p16 in the fibroblasts. The expression of p21 and p16 in the control fibroblast group was lower than that in each of the H_2_O_2_-treated fibroblast groups. Among the H_2_O_2_-treated fibroblast groups, the expression of p21 and p16 was lowest in the H_2_O_2_/PDLLA-CM_ASCs_-treated group (Figure S14).

### 3.7. PDLLA Reduced Fibroblast Senescence by Modulating Macrophages

We evaluated the ability of PDLLA to attenuate fibroblast senescence by modulating macrophages in an in vitro model. PBS/PBS-CM_MΦ_ was administered to PBS-treated fibroblasts (control fibroblasts). LPS/PBS-CM_MΦ_, LPS/PDLLA-CM_MΦ_, or LPS/HA-CM_MΦ_ were administered to H_2_O_2_-treated fibroblasts (Figure S7). Because we treated the fibroblasts with a mixture of growth medium and conditioned media, we first confirmed that the expression of the fibroblast marker vimentin was not changed by the mixture of growth medium and conditioned media compared with growth medium alone (Figure S15). Measurement of the senescence markers p21 and p16 showed that control fibroblasts treated with PBS/PBS-CM_MΦ_ had lower p21 and p16 expression than each of the H_2_O_2_-treated fibroblast groups. Among the H_2_O_2_-treated fibroblast groups, the expression of p21 and p16 was lowest in the group treated with LPS/PDLLA-CM_MΦ_ (Figure S16).

### 3.8. PDLLA-Induced Modulation of ASCs Led to Increased Proliferation and Collagen Synthesis in Senescent Fibroblasts

The proliferation ratio of control fibroblasts was higher than that of H_2_O_2_-treated fibroblasts. Among the H_2_O_2_-treated fibroblast groups, the proliferation ratio of the group treated with H_2_O_2_/PDLLA-CM_ASCs_ was higher than those of the groups treated with H_2_O_2_/PBS-CM_ASCs_ (Figure 3D).

To evaluate collagen fiber synthesis, we measured collagen 1a1 (COL1a1) and collagen 3a1 (COL3a1) by ELISA. The expression of COL1a1 and COL3a1 in control fibroblasts was higher than that in each of the H_2_O_2_-treated fibroblast groups. Among the H_2_O_2_-treated fibroblast groups, the group treated with H_2_O_2_/PDLLA-CM_ASCs_ had higher COL1a1 and COL3a1 expression than the groups treated with H_2_O_2_/PBS-CM_ASCs_ or H_2_O_2_/HA-CM_ASCs_ (Figure 3E,F).

To evaluate elastin fiber synthesis, we measured the expressions of elastin (ELN) and elastin-binding protein (EBP) by ELISA. The expression of EBP and ELN in control fibroblasts was higher than that in each of the H_2_O_2_-treated fibroblast groups. Among the H_2_O_2_-treated fibroblast groups, the group treated with H_2_O_2_/PDLLA-CM_ASCs_ had higher EBP expression than those treated with H_2_O_2_/PBS-CM_ASCs_. The expression of ELN was higher in the group treated with H_2_O_2_/PDLLA-CM_ASCs_ than in the group treated with H_2_O_2_/PBS-CM_ASCs_ or H_2_O_2_/HA-CM_ASCs_ (Figure 3G,H).

### 3.9. PDLLA-Induced Modulation of Macrophage Led to Increased Proliferation and Collagen Synthesis in Senescent Fibroblasts

The proliferation ratio of control fibroblasts was higher than that of H_2_O_2_-treated fibroblasts. Among the H_2_O_2_-treated fibroblast groups, the proliferation ratio of the group treated with LPS/PDLLA-CM_MΦ_ was higher than those of the groups treated with LPS/PBS-CM_MΦ_ (Figure 3I).

The expression of COL1a1 and COL3a1 in control fibroblasts was higher than that in each of the H_2_O_2_-treated fibroblast groups. Among the H_2_O_2_-treated fibroblast groups, the group treated with LPS/PDLLA-CM_MΦ_ had higher COL1a1 and COL3a1 expression than the groups treated with LPS/PBS-CM_MΦ_ (Figure 3J,K).

The expression of EBP and ELN in control fibroblasts was higher than that in each of the H_2_O_2_-treated fibroblast groups. Among the H_2_O_2_-treated fibroblast groups, the group treated with LPS/PDLLA-CM_MΦ_ had higher EBP and ELN expression than those treated with LPS/PBS-CM_MΦ_ (Figure 3L,M).

### 3.10. PDLLA Increased Fibroblast Proliferation and Collagen Synthesis in Aged Skin

**Figure 4:**
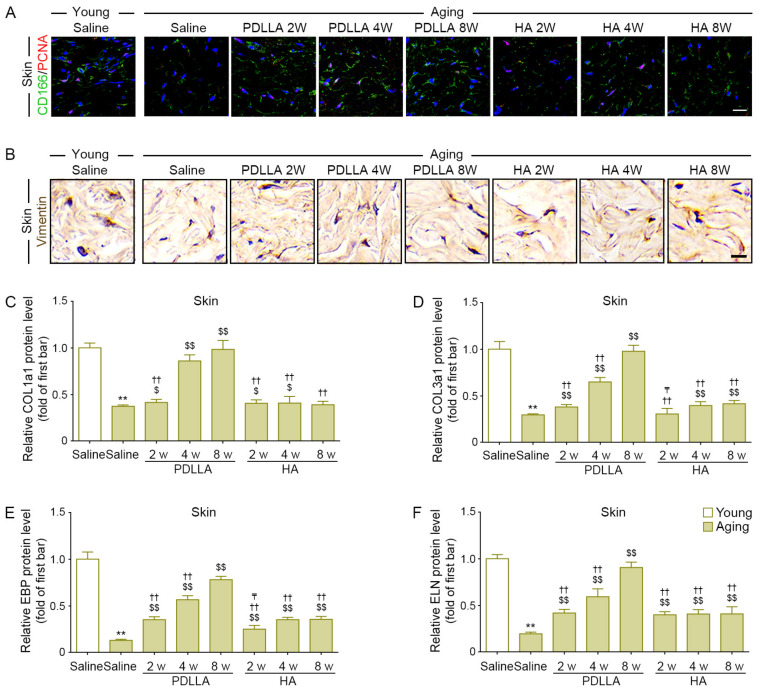
Increased ASCs proliferation in aged skin modulated by PDLLA led to increased dermal fibroblast proliferation and collagen/elastin synthesis. (**A**) Colocalization of CD166 (ASCs marker; green) and PCNA (proliferation marker; red) was assessed by immunofluorescence (nuclei; blue) (scale bar = 50 μm). ASCs proliferation was lower in saline-injected aged skin compared with that in saline-injected young skin and was increased in aged skin by PDLLA or HA injection, except at 8 weeks after injection. (**B**) The expression levels of vimentin (fibroblast marker) in aged skin were assessed using DAB staining (scale bar = 50 μm). The expression of vimentin was reduced in aged skin compared with that in young skin and was increased in aged skin by PDLLA or HA. (**C**–**F**) The protein expression of COL1a1 (**C**), COL3a1 (**D**), EBP (**E**), and ELN (**F**) in aged skin was assessed by ELISA. The expression of COL1a1 (**C**), COL3a1 (**D**), EBP (**E**), and ELN (**F**) was reduced in aged skin compared with that in young skin and was increased in aged skin by PDLLA or HA. The data were represented relative to the mean of the first bar of the graph. Data are presented as the mean ± SD (*n* = 3/group). **, *p* < 0.01, first bar vs. second bar; $ and $$, *p* < 0.05 and *p* < 0.01, second bar vs. third bar~eighth bar; ††, *p* < 0.01, fifth bar vs. third bar~eighth bar; ₸, *p* < 0.05, eighth bars vs. sixth, seventh bar.

We performed proliferating cell nuclear antigen (PCNA) staining to evaluate ASCs proliferation in the dermis. The intensity of PCNA colocalization with CD166, an ASCs marker, was lower in saline-injected aged skin than in saline-injected young skin at 8 weeks after injection. The intensity of PCNA colocalization with CD166 in saline-injected aged skin at 2 and 4 weeks after injection was higher than that in PDLLA- or HA-injected aged skin at 8 weeks after injection; however, it was not significantly different from that in PDLLA- or HA-injected aged skin at 8 weeks after injection (Figure 4A, Figures S17 and S18A).

We performed vimentin staining to evaluate fibroblast density. The expression of vimentin in saline-injected aged skin was lower than that in saline-injected young skin at 8 weeks after injection. The expression of vimentin in saline-injected aged skin at 8 weeks after injection was lower than that in PDLLA- or HA-injected aged skin at 2, 4, and 8 weeks after injection. Among the PDLLA- and HA-injected groups, the vimentin expression was highest in PDLLA-injected aged skin at 8 weeks after injection (Figure 4B and Figure S18B).

COL1a1 and COL3a1 levels were lower in saline-injected aged skin than in saline-injected young skin at 8 weeks after injection. The COL1a1 and COL3a1 levels in PDLLA-injected aged skin at 2, 4, and 8 weeks after injection were higher than those in saline-injected aged skin. The COL1a1 levels in HA-injected aged skin at 2 and 4 weeks after injection were higher than those in saline-injected aged skin, whereas the COL1a3 levels in HA-injected aged skin at 4 and 8 weeks after injection were higher than those in saline-injected aged skin. Among the PDLLA- or HA-injected groups, the COL1a1 and COL3a1 levels were highest in the PDLLA-injected aged skin at 8 weeks after injection (Figure 4C,D).

EBP and ELN levels were lower in saline-injected aged skin than in saline-injected young skin at 8 weeks after injection. The EBP and ELN levels were higher in PDLLA- or HA-injected aged skin at 2, 4, and 8 weeks after injection than in saline-injected aged skin. Among the PDLLA- or HA-injected groups, the EBP and ELN levels were highest in the PDLLA-injected aged skin at 8 weeks after injection (Figure 4E,F).

### 3.11. PDLLA Reduced the Expression of NF-κB and MMPs in Senescent Fibroblasts by Modulating Macrophages and ASCs

**Figure 5:**
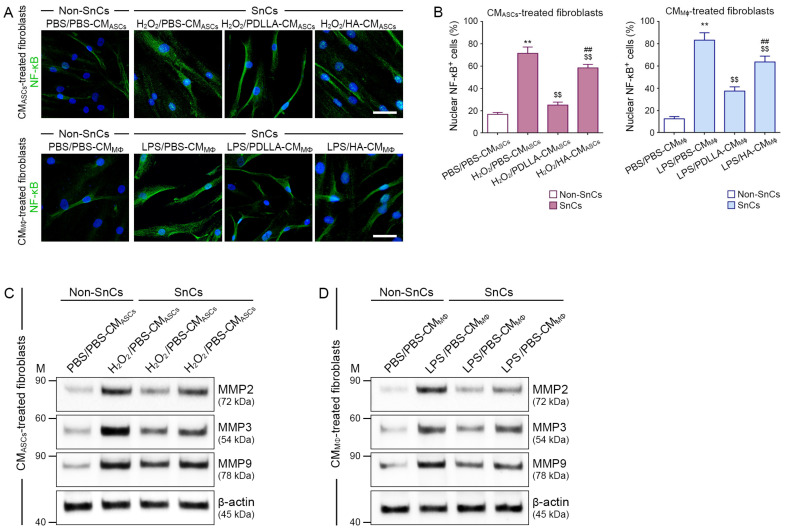
Reduced NF-κB and MMPs expression in senescent fibroblasts by PDLLA via effects on macrophages and ASCs. (**A**) The effects of macrophage-modulated ASCs on NF-κB expression in fibroblasts were measured by immunocytochemistry (upper channel). The expression of NF-κB in macrophage-influenced senescent fibroblasts was validated by immunocytochemistry (lower channel; green: positive signals; blue: nuclei) (scale bar = 20 μm). (**B**) These graphs quantify the data in (**A**). The percentage of nuclear NF-κB positive cells was higher in senescent fibroblasts (SnCs) treated with H_2_O_2_/PBS-CM_ASCs_ or LPS/PBS-CM_MΦ_ than in control fibroblasts (non-senescent cells, non-SnCs). The increase in the percentage of nuclear NF-κB positive cells was attenuated in senescent fibroblasts treated with H_2_O_2_/PDLLA-CM_ASCs_, H_2_O_2_/HA-CM_ASCs,_ or LPS/PDLLA-CM_MΦ_, LPS/HA-CM_MΦ_. The data were represented relative to the mean of the first bar of the graph. (**C**,**D**) The protein expression of MMP2, MMP3, and MMP9 in senescent fibroblast was validated by Western blot. The expression of MMP2, MMP3, and MMP9 was higher in fibroblasts treated with H_2_O_2_/PBS-CM_ASCs_ or LPS/PBS-CM_MΦ_ than in control fibroblasts. The increase in MMP2, MMP3, and MMP9 expression was attenuated in fibroblasts treated with H_2_O_2_/PDLLA-CM_ASCs_, H_2_O_2_/HA-CM_ASCs_ or LPS/PDLLA-CM_MΦ_, LPS/HA-CM_MΦ_. To correct for differences in protein loading, the quantification of the Western blot was normalized using β-actin as a loading control protein. Data are presented as the mean ± SD (*n* = 3/group). **, *p* < 0.01, first bar vs. second bar; $$, *p* < 0.01, second bar vs. third bar or fourth bar; ##, *p* < 0.01, third bar vs. fourth bar.

We evaluated the ability of ASCs modulated by macrophages to reduce NF-κB and MMP levels in H_2_O_2_-treated fibroblasts. The NF-κB expression in control fibroblasts was lower than that in each of the H_2_O_2_-treated fibroblast groups. The expression of NF-κB in fibroblasts treated with H_2_O_2_/PBS-CM_ASCs_ was higher than that in fibroblasts treated with H_2_O_2_/PDLLA-CM_ASCs_ or H_2_O_2_/HA-CM_ASCs_. The fibroblasts treated with H_2_O_2_/PDLLA-CM_ASCs_ had lower NF-κB expression than those treated with H_2_O_2_/HA-CM_ASCs_ (Figure 5A,B). The expression of MMP2/3/9 in control fibroblasts was lower than that in each H_2_O_2_-treated fibroblast group. Fibroblasts treated with H_2_O_2_/PBS-CM_ASCs_ had higher expression of MMP2/3/9 than fibroblasts treated with H_2_O_2_/PDLLA-CM_ASCs_ or H_2_O_2_/HA-CM_ASCs_. The expression of MMP2/3/9 in fibroblasts treated with H_2_O_2_/PDLLA-CM_ASCs_ was lower than that in fibroblasts treated with H_2_O_2_/HA-CM_ASCs_ (Figure 5C and Figure S19A–C).

Next, we evaluated whether macrophages modulated by PDLLA could directly affect fibroblasts to reduce the expression of NF-κB and MMP2/3/9. The expression of NF-κB in control fibroblasts was lower than that in each of the H_2_O_2_-treated fibroblast groups. Fibroblasts treated with LPS/PBS-CM_MΦ_ had higher NF-κB expression than fibroblasts treated with LPS/PDLLA-CM_MΦ_ or LPS/HA-CM_MΦ_. The expression of NF-κB was lower in fibroblasts treated with LPS/PDLLA-CM_MΦ_ than in fibroblasts treated with LPS/HA-CM_MΦ_ (Figure 5A,B). The expression of MMP2/3/9 in the control fibroblast was lower than that in each of the H_2_O_2_-treated fibroblast groups. Fibroblasts treated with LPS/PBS-CM_MΦ_ had higher MMP2/3/9 expression than fibroblasts treated with LPS/PDLLA-CM_MΦ_ or LPS/HA-CM_MΦ_. The expression of MMP2/3/9 was lower in fibroblasts treated with LPS/PDLLA-CM_MΦ_ than in fibroblasts treated with LPS/HA-CM_MΦ_ (Figure 5D and Figure S19D–F).

### 3.12. PDLLA Reduced the Expression of NF-κB and MMPs in Aged Skin

**Figure 6:**
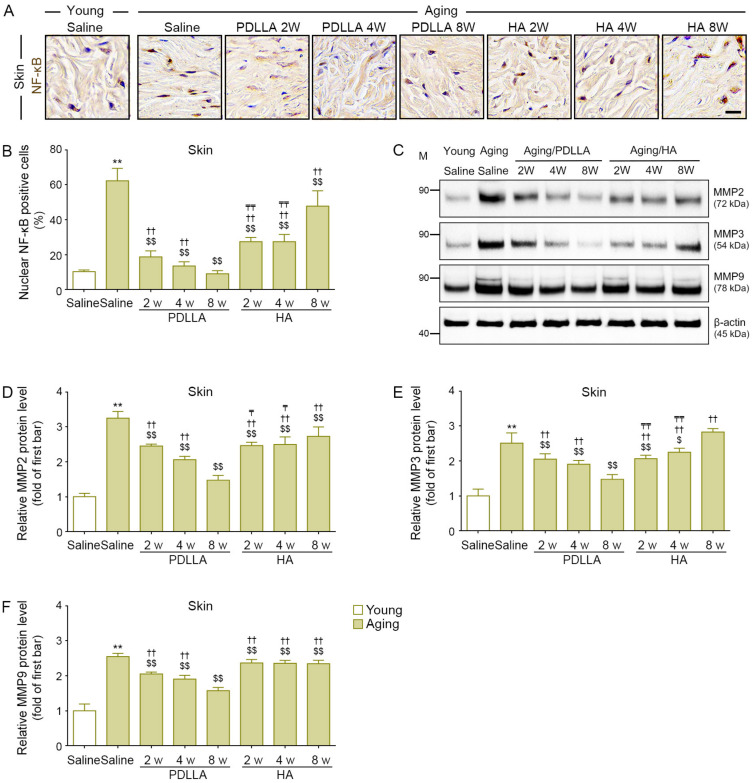
Reduced NF-κB and MMPs expression by PDLLA in aged skin. (**A**) The expression levels of NF-κB in aged skin were validated by DAB staining (scale bar = 50 μm). (**B**) This graph quantifies the data in (**A**). The percentage of nuclear NF-κB positive cells was increased in aged skin compared with that in young skin. The increased NF-κB expression in aged skin was attenuated by PDLLA or HA. (**C**) The protein expression of MMP2, MMP3, and MMP9 in aged skin was validated by Western blot. (**D**–**F**) This graph quantifies the data in (**C**). The expression of MMP2, MMP3, and MMP9 was increased in aged skin compared with that in young skin. The increased MMP2, MMP3, and MMP9 expression in aged skin was attenuated by PDLLA or HA. To correct for differences in protein loading, the quantification of the Western blot was normalized using β-actin as a loading control protein. For each blot, the values were expressed relative to the mean of the first bar in the graph. Data are presented as the mean ± SD (*n* = 3/group). **, *p* < 0.01, first bar vs. second bar; $ and $$, *p* < 0.05 and *p* < 0.01, second bar vs. third bar~eighth bar; ††, *p* < 0.01, fifth bar vs. third bar~eighth bar; ₸ and ₸₸, *p* < 0.05 and *p* < 0.01, eighth bars vs. sixth, seventh bar.

The expression of NF-κB in saline-injected aged skin was higher than that in saline-injected young skin at 8 weeks after injection. Among the aged-skin groups, the expression of NF-κB in the saline-injected group at 8 weeks after injection was higher than that in the PDLLA- or HA-injected groups at 2, 4, and 8 weeks after injection. The NF-κB expression in PDLLA-injected skin was lowest at 8 weeks after injection; however, the NF-κB expression in HA-injected skin was highest at 8 weeks after injection (Figure 6A,B).

The expression of MMP2/3/9 in saline-injected aged skin was higher than that in saline-injected young skin at 8 weeks after injection. Among the aged-skin groups, the expression of MMP2/3/9 in the saline-injected group was higher than that in the PDLLA- or HA-injected groups at 2, 4, and 8 weeks after injection. In PDLLA-injected skin, MMP2/3/9 levels were lowest at 8 weeks after injection. In HA-injected skin, MMP2/3 levels were lowest at 2 weeks after injection. There was no significant difference in MMP9 levels in HA-injected skin at 2, 4, and 8 weeks after injection (Figure 6C–F).

### 3.13. PDLLA Increased the Expression of Collagen and Elastin Fibers in Aged Skin

**Figure 7:**
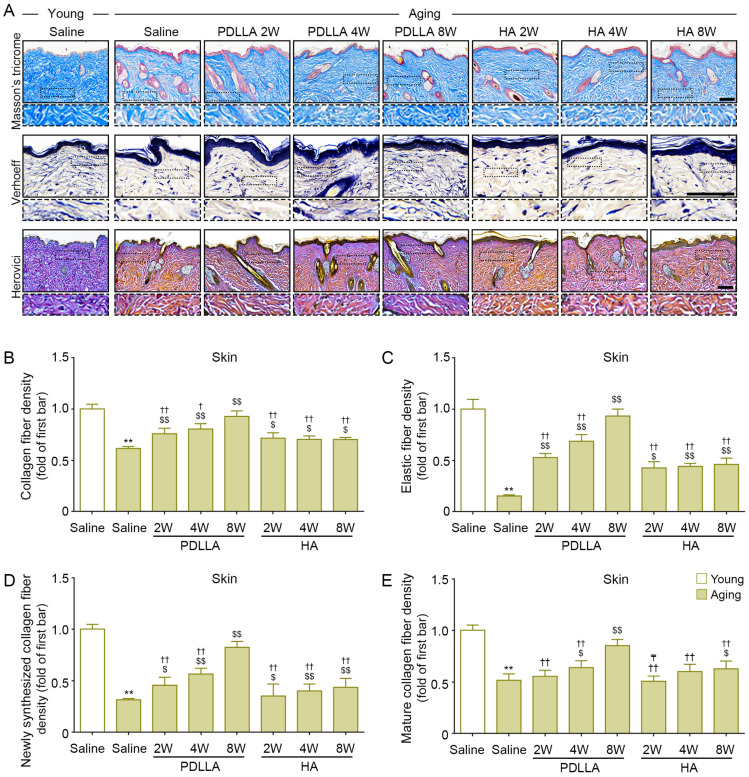
Upregulated collagen and elastin fibers by PDLLA in aged skin. (**A**) Masson’s trichrome, Verhoeff’s Gieson, and Herovici’s staining in aged skin (scale bar = 100 µm). (**B–E**) This graph quantifies the data in (**A**). The density of collagen fibers (**B**), elastin fibers (**C**), newly synthesized collagen fibers (**D**), and mature collagen fibers (**E**) was lower in aged skin than in young skin and was increased in aged skin by PDLLA or HA. The data were represented relative to the mean of the first bar of the graph. Data are presented as the mean ± SD (*n* = 3/group). **, *p* < 0.01, first bar vs. second bar; $ and $$, *p* < 0.05 and *p* < 0.01, second bar vs. third bar~eighth bar; † and ††, *p* < 0.05 and *p* < 0.01, fifth bar vs. third bar~eighth bar; ₸ *p* < 0.05, eighth bars vs. sixth or seventh bar.

The expression of collagen fiber was evaluated with Masson’s trichrome stain. The collagen fiber density of saline-injected aged skin was lower than that of saline-injected young skin at 8 weeks after injection. Among the aged-skin groups, the collagen fiber density was lower in the saline-injected group than in the PDLLA- or HA-injected groups at 2, 4, and 8 weeks after injection. In PDLLA-injected skin, the collagen density increased over time; however, it did not increase over time in HA-injected skin (first row of Figure 7A,B).

The expression of elastin, the main component of the skin ECM, was evaluated by Verhoeff staining. The expression of elastin fibers in saline-injected aged skin was lower than that in saline-injected young skin at 8 weeks after injection. Saline-injected aged skin had lower elastin fiber expression than PDLLA- or HA-injected aged skin at 2, 4, and 8 weeks after injection. In PDLLA-injected skin, the expression of elastin increased over time; however, it did not increase over time in HA-injected skin (second row of Figure 7A,C).

Herovici collagen staining can differentiate newly formed collagen (stained blue) from mature collagen (stained red) [51,52]. The expression of newly synthesized collagen in saline-injected aged skin was significantly lower than that in saline-injected young skin at 8 weeks after injection. The expression of newly synthesized collagen in saline-injected aged skin was lower than that in PDLLA- or HA-injected aged skin at 2, 4, and 8 weeks after injection. The expression of newly synthesized collagen increased over time in both PDLLA-injected skin and HA-injected skin (third row of Figure 7A,D). The expression of mature collagen in saline-injected aged skin was lower than that in saline-injected young skin at 8 weeks of injection. The expression of mature collagen in saline-injected aged skin was lower than that in PDLLA-injected aged skin at 2, 4, and 8 weeks after injection and lower than that in HA-injected skin at 8 weeks after injection. The expression of mature collagen increased over time in both PDLLA-injected skin and HA-injected skin (third row of Figure 7A,E).

## 4. Discussion

Dermal fillers can attenuate aging-induced loss of soft tissue volume. HA injection has various biologic effects including tissue hydration [53] and antioxidant activity [54,55]. HA also decreases apoptosis and promotes cell migration during wound healing [56,57,58] and increases fibroblast proliferation and synthesis of type I collagen [53]. One way that injection of HA filler induces collagen synthesis is by generating mechanical stress leading to fibroblast stretching [59,60]. Fibroblasts at the site of dermal HA injection show an elongated and stretched appearance and express elevated levels of type I procollagen [59]; however, this mechanically stimulated collagen synthesis is only maintained for a relatively short period [61].

The polymer microspheres in PLLA dermal filler have additional biological effects that trigger collagen synthesis [62]. After injection of PLLA filler, immune cells, such as macrophages, lymphocytes, and giant cells, coat the microspheres, resulting in a mild inflammatory reaction. The immune cells promote fibroblast migration to the microspheres and induce collagen synthesis, which leads to capsule formation around the microspheres. After 6 months, the capsules gradually disappear and are replaced by newly synthesized collagen [63,64]. The microspheres are eventually hydrolyzed into water and carbon dioxide [62]. PDLLA is a chiral variant of PLLA. PDLLA and PLLA have the same chemical formula but generate differently shaped microspheres [37]. PLLA microspheres are irregular solid structures, whereas PDLLA microspheres have a sponge-like structure [12,14]. Because the PDLLA microspheres have pores, they not only stimulate collagen synthesis but also act as three-dimensional scaffolds for neo-tissue generation [37,65].

The lactic acid that exists in organisms is L-type, so L-lactic acid is thought to be more biocompatible than D-lactic acid [66]. An animal study showed that PLLA microspheres caused less inflammation and more collagen synthesis than PDLLA microspheres [66]. However, a multicenter randomized clinical trial that evaluated safety for 24 months found that PDLLA filler did not cause serious complications [67]. Some studies reported that PDLLA has a shorter reconstitution time and lower requirement for reconstitution water than PLLA [68]. Furthermore, complications such as nodule formation are less reported for PDLLA than for PLLA [68]. Because PDLLA has a porous structure, the total volume of a given weight of PDLLA filler is much higher than that of the same weight of PLLA filler, which results in a greater early-stage volume-restoring effect for PDLLA [68].

Immune cells play essential roles in collagen synthesis. Despite that, the function of macrophages after PLA filler injection has been studied only with regard to the mild foreign body reaction to initiate fibroblast migration and proliferation [62,69,70]. During aging, various skin cells are chronically exposed to mild inflammation and oxidative stress [71]. Compared with younger macrophages, senescent macrophages release greater amounts of MMPs and ROS, which aggravate inflammation [72]. Furthermore, senescent dermal fibroblasts secrete more pro-inflammatory cytokines and synthesize less collagen than younger fibroblasts [71]. Hence, the mechanism by which dermal fillers increase collagen synthesis in aged skin cannot be fully explained by the fact that PLA microspheres stimulate immune cells to modulate fibroblast collagen synthesis. Previous studies that evaluated the effects of PLA filler used mainly young animals and showed that PLA filler increased dermal thickness and collagen fiber density [73]. Because collagen synthesis is reduced by cellular senescence, we hypothesized that modulation of cellular senescence would enhance the effect of dermal fillers on collagen synthesis.

Interest in ASCs as a skin rejuvenating or wound healing modality has rapidly increased. Conditioned media from ASCs are known to contain various cytokines such as epidermal growth factor (EGF), vascular growth factor (VEGF), platelet-derived growth factor (PDGF)-AA, and FGF, which promote fibroblast migration and proliferation [74]. These paracrine factors from ASCs may also promote collagen synthesis, increase skin elasticity, and attenuate skin aging [22]. Pre-treatment with ASCs-conditioned media before exposure to UV-B irradiation reduced senescence and increased expression of type I and III collagen in dermal fibroblasts [75]. ASCs also reduce the expression of MMP1/9 and attenuate UV-B-induced photoaging [76]. We hypothesized that ASCs would play an important role in the fibroblast stimulation induced by PDLLA in aged skin.

The role of NRF2 in skin aging has been widely examined; however, the role of NRF2 in PDLLA-induced collagen synthesis has not been evaluated. We focused on NRF2 expression in macrophages, which are the initiator cells of serial reactions following dermal filler injection. First, we assessed the expression of the senescence markers p21 and p16 in H_2_O_2_-treated macrophages. We found that PDLLA reduced p21 and p16 expression in the senescence-induced macrophages. NRF2 levels decrease during aging, so we also evaluated whether PDLLA restores NRF2 activity in senescent macrophages. In homeostatic conditions, NRF2 stays in the cytoplasm by binding with Kelch-like ECH-associated protein-1 (Keap1) which facilitates the degradation of NRF2 [77]. However, NRF2 is released from Keap1 and further translocated to the nucleus by NRF2 activating signals [77]. Phosphorylation at Ser40 of NRF2 leads to nuclear translocation of NRF2 and increases its transcriptional activity [77].

Activation of NRF2 is known to reduce oxidative stress and inflammation by inhibiting various signaling pathways such as NF-κB and NAD(P)H oxidase 4 [78,79]. Increased nuclear translocation of NRF2 also inhibits macrophage polarization toward the M1 phenotype [80]. Our results with in vitro models showed that PDLLA increases NRF2 phosphorylation at Ser40, M2 macrophage polarization, and IL-10 expression in senescent macrophages.

IL-10 is known to promote stem cell survival, so we evaluated the ability of IL-10 secreted from PDLLA-stimulated macrophages to modulate ASCs function. Conditioned media from senescent macrophages increased p21 and p16 expression in senescent ASCs; however, when the senescent macrophages were treated with PDLLA, their conditioned media reduced p21 and p16 in senescent ASCs. This suggests that PDLLA reduced ASCs senescence by modulating the macrophages to increase their IL-10 secretion. PDLLA treatment of senescent macrophages also increased the proliferation and paracrine function of senescent ASCs, which secrete TGF-β and FGF.

Next, we evaluated the paracrine effects of ASCs on fibroblast collagen synthesis. We hypothesized that PDLLA first modulates macrophages, which in turn modulate ASCs, and the ASCs ultimately modulate fibroblasts. We treated senescent ASCs with conditioned media from PDLLA-treated senescent macrophages. We then collected conditioned media from the ASCs and administered it to senescent fibroblasts. These in vitro studies showed that PDLLA modulates senescent macrophages and ASCs, eventually leading to increased proliferation and collagen and elastin synthesis in senescent fibroblasts.

We also evaluated whether modulated macrophage by PDLLA could directly affect fibroblast senescence and collagen synthesis ability not via the action of ASC. Expression of the senescence markers p21 and p16 of fibroblast were attenuated by treating conditioned media from PDLLA or HA-treated macrophage. The proliferation ratio of fibroblast and expression of COL1a1, COL3a1, EBP, and ELN were decreased in the fibroblast by treating conditioned media from the senescent macrophage. Those were attenuated by treating conditioned media from PDLLA or HA-treated macrophage. Those results suggest that macrophage modulated by PDLLA also directly decreased fibroblast senescence and increased collagen synthesis without ASCs.

During the aging process, increased oxidative stress leads to upregulation of NF-κB, which eventually increases the expression of MMPs [81,82]. Transcriptome analysis of human skin showed that the NF-κB signaling pathway changes during aging [83]. Furthermore, genetic deletion of NF-κB in aged animals resulted in skin rejuvenation [84]. Because increased NRF2 levels can reduce the expression of NF-κB, we evaluated the ability of PDLLA-induced NRF2 activity in macrophages to reduce NF-κB and MMPs expression in fibroblasts. When we cultured senescent fibroblasts with conditioned media from PDLLA-treated senescent macrophages, the conditioned media reduced fibroblast senescence and expression of NF-κB and MMPs. These results suggest that PDLLA can attenuate ECM destruction by modulating macrophages to reduce the expression of NF-κB and MMPs in fibroblasts. Moreover, we evaluated whether macrophage modulation of ASCs resulted in decreased expression of NF-κB and MMPs in the senescent fibroblast. Expression of NF-κB and MMPs was decreased by conditioned media from the ASCs which had been administered with conditioned media from PDLLA-treated senescent macrophage. Those results showed that PDLLA-modulated macrophage decreased expression of NF-κB and MMPs in the fibroblast via ASCs or directly.

In our in vitro models, the beneficial effects of PDLLA on macrophages, ASCs, and fibroblasts were stronger than those of HA. HA filler has immunosuppressive and anti-inflammatory properties [85]. Its action to increase collagen synthesis is due to the mechanical stimulation of fibroblasts rather than the biological mediation of inflammation [59]. Our results show that HA increases IL-10, M2, and NRF2 expression in macrophages, but to a significantly lesser degree than PDLLA.

Although the best-known mechanism for PLA filler to stimulate collagen synthesis involves immune cells such as macrophages, many in vitro studies of the effects of PLA filler on collagen synthesis use fibroblast cell lines stimulated directly with PLA [86]. We thought that this approach is not the best way to evaluate immune cell action, so we used a series of in vitro models with conditioned media. Our results suggest that PDLLA reduces fibroblast senescence by modulating macrophages. PDLLA also reduces ASCs senescence by reducing macrophage’s senescence, which eventually leads to increased ASCs paracrine effects. The increased ASCs paracrine effects in turn increase the ability of senescent fibroblasts to proliferate and synthesize collagen.

The patterns of in vitro results were consistent with the results from our animal model. Aged animal skin exhibited reduced expression of NFR2, M2, and IL-10, which was attenuated by PDLLA injection. PDLLA also increased the rate of ASCs proliferation, which was low in aged skin. Furthermore, the low fibroblast density and expression of COL1a1, COL3a1, EBP, and ELN in aged skin were all increased by PDLLA. Conversely, the expression of NF-κB and MMPs was high in aged skin and was decreased by PDLLA. PDLLA also increased the low density of collagen and elastin fibers in the dermis of aged skin. The expression of COL1a1, COL3a1, EBP, and ELN was increased by PDLLA over time and was highest at 8 weeks after PDLLA injection. The expression of IL-10 was also highest at 8 weeks after PDLLA injection; however, ASCs proliferation was highest at 4 weeks after the injection. Collagen fiber density was highest at 8 weeks after PDLLA injection, whereas expression of NF-κB and MMPs was lowest at 8 weeks after the injection. It is known that IL-10 decreases NF-κB expression in dermal fibroblasts [87]. Although ASCs proliferation was maximized at 4 weeks after PDLLA injection, IL-10 continuously reduced NF-κB expression, which in turn reduced MMPs levels and attenuated the age-related destruction of collagen fibers. The increased collagen synthesis and decreased collagen destruction by MMPs were more prominent in PDLLA-injected aged skin than in HA-injected aged skin. The volume-increasing effect of PDLLA is known to be maintained until 20 weeks after injection [68]. This gives PDLLA an advantage over HA because its volume effect lasts longer and it also stimulates collagen synthesis by modulating immune cells [69,70,88].

## 5. Conclusions

**Figure 8:**
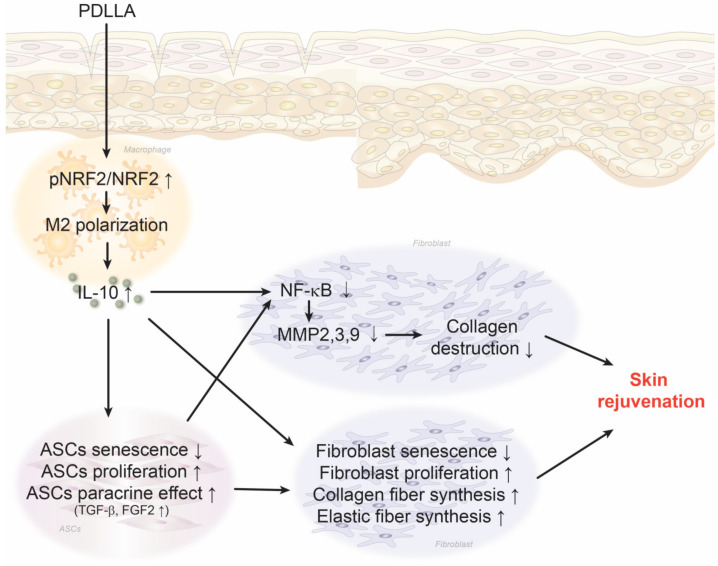
Summary of this study. PDLLA enhances pNRF2/NRF2 expression in macrophages, which leads to increased M2 polarization and IL-10 expression in senescent macrophages and aged skin. This increase in IL-10 expression by macrophages leads to reduced senescence and increased proliferation and secretion of TGF-β and FGF2 in ASCs. The increase in TGF-β and FGF2 levels results in increased proliferation and synthesis of collagen and elastin fibers in fibroblasts. The PDLLA-modulated macrophage not only directly stimulated fibroblast activity, promoting proliferation and collagen synthesis, but also reduced the expression of NF-κB and MMP2/3/9. These effects ultimately led to skin rejuvenation in aged skin.

PDLLA increased macrophage NRF2 expression, resulting in increased M2 polarization and IL-10 expression in senescent macrophages and aged skin. The increased IL-10 expression by macrophages led to reduced ASCs senescence and increased ASCs proliferation and paracrine secretion of TGF-β and FGF2. The increased TGF-β and FGF2 levels in turn led to increased fibroblast proliferation and synthesis of collagen and elastin fibers. Macrophage modulated by PDLLA also directly stimulated fibroblast to increase proliferation and collagen synthesis. PDLLA also decreased the expression of NF-κB and MMP2/3/9 which eventually resulted in skin rejuvenation in aged skin (Figure 8).

## Data Availability

Data is contained within the article and Supplementary Materials.

## Supplementary Materials

The following supporting information can be downloaded at: https://www.mdpi.com/article/10.3390/antiox12061204/s1, Figure S1: The morphology and size of PDLLA particles; Figure S2: Concentration of H2O2 for establish a senescence model of macrophages; Figure S3: The optimal concentration of PDLLA or HA to reduce senescence in senescent macrophages; Figure S4: Schematic diagram to confirm the efficacy of PDLLA in senescent macrophages; Figure S5: Schematic diagram to confirm the regulation of senescent ASCs proliferation and paracrine secretion by PDLLA-treated senescent macrophages; Figure S6: Schematic diagram to confirm the modulation of senescent fibroblast proliferation and collagen synthesis by PDLLA-CMMΦ-treated senescent ASCs; Figure S7: Schematic diagram to confirm the modulation of senescent fibroblast proliferation and collagen synthesis by PDLLA-treated senescent macrophages; Figure S8: The reduction effect of senescence in H2O2-induced senescence murine macrophages by PDLLA treatment; Figure S9: Upregulated pNRF2 activation and M2 polarization by PDLLA or HA treatment in senescent macrophages; Figure S10: The reduction effect of senescence in senescent macrophages by PDLLA treatment; Figure S11: Changes in the character of ASCs upon treatment with a mixture of CMMΦ and growth medium; Figure S12: Reduced effect of senescence on ASCs via modulation of macrophages treated with PDLLA; Figure S13: Changes in the character of fibroblasts upon treatment with CMASCs and growth medium mixture; Figure S14: Reduced effect of senescence on fibroblast via modulation of ASCs affected with PDLLA. PDLLA modulation of ASCs reduced aging effects in H2O2-induced senescent fibroblasts; Figure S15: The CMMΦ and growth medium mixture did not change the character of fibroblasts; Figure S16: Reduced effect of senescence on fibroblasts via modulation of macrophages treated with PDLLA; Figure S17: Upregulatory effects of PDLLA on ASCs proliferation in aged skin; Figure S18: Upregulatory effects of PDLLA on dermal fibroblast proliferation in aged skin; Figure S19: Reduced MMPs expression in senescent fibroblasts by PDLLA via effects on macrophages and ASCs; Table S1: List of primer for qRT-PCR in this study; Table S2: List of antibodies used in this study.
