# Supplementary material for: Poly-D,L-Lactic Acid Filler Increases Extracellular Matrix by Modulating Macrophages and Adipose-Derived Stem Cells in Aged Animal Skin
Source: Antioxidants (Basel). 2023 Jun 1;12(6):1204. doi: 10.3390/antiox12061204 (PMC10294940; doi:10.3390/antiox12061204)
Supplement: Supplementary file 1 [file antioxidants-12-01204-s001.zip › antioxidants-2374471-supplementary.pdf]

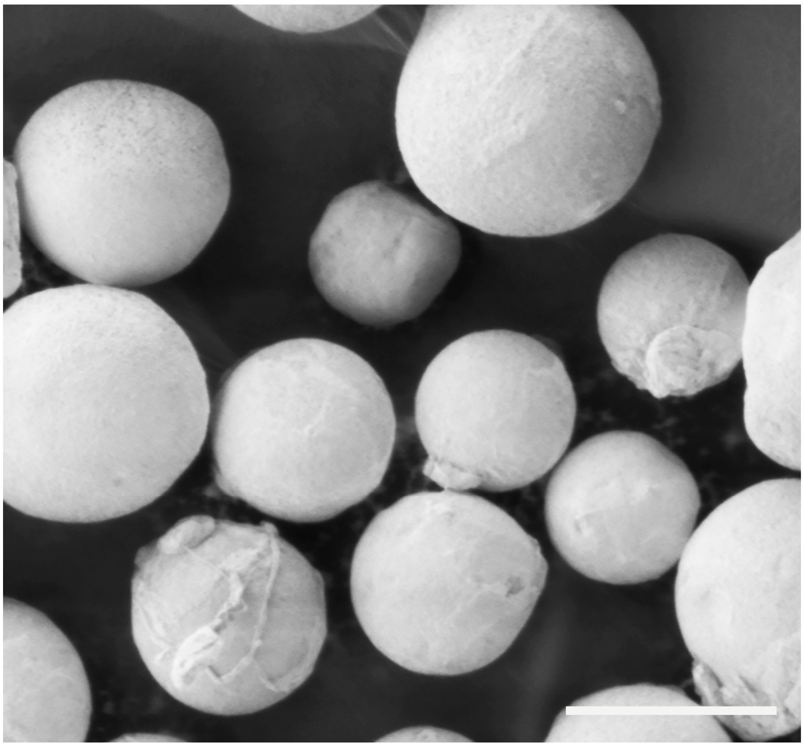

Figure S1. The morphology and size of PDLLA particles. The PDLLA particles have a size of 10–30 μm (Scale bar = 20 μm).

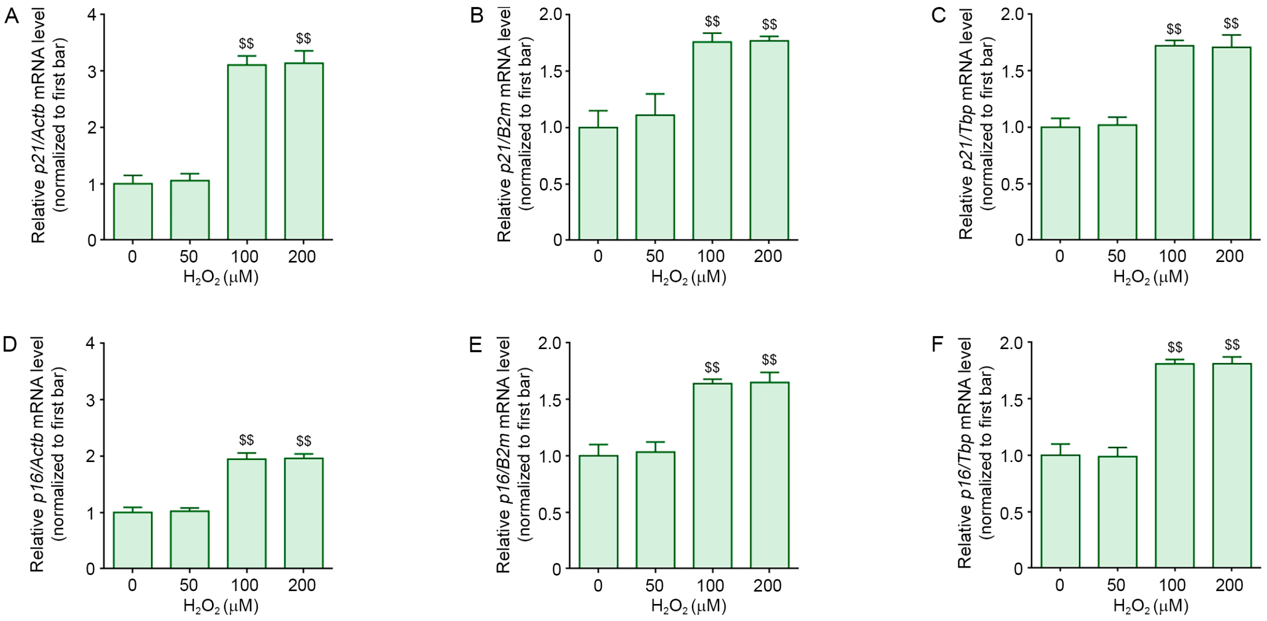

Figure S2. The concentration of H_2O_2 to establish a senescence model of macrophages. (**A–F**) To obtain the optimal H_2O_2 concentration to induce macrophage senescence (RAW 264.7), the mRNA expression of p21 and p16 (senescence markers) was measured by qRT-PCR. The mRNA expression of p21 and p16 showed no change at 50 μM H_2O_2 and increased at 100 μM H_2O_2. There was no further increase at 200 μM H_2O_2, so 100 μM was determined to be the optimal H_2O_2 concentration to induce macrophage senescence. Data were normalized to Actb, B2m, or Tbp, and expression levels were reported according to the comparative CT method (ΔΔCT) relative to the first bar in the graph. Data are presented as the mean ± SD (n = 3/group). $$, $p < 0.01$, second bar vs. third bar or fourth bar.

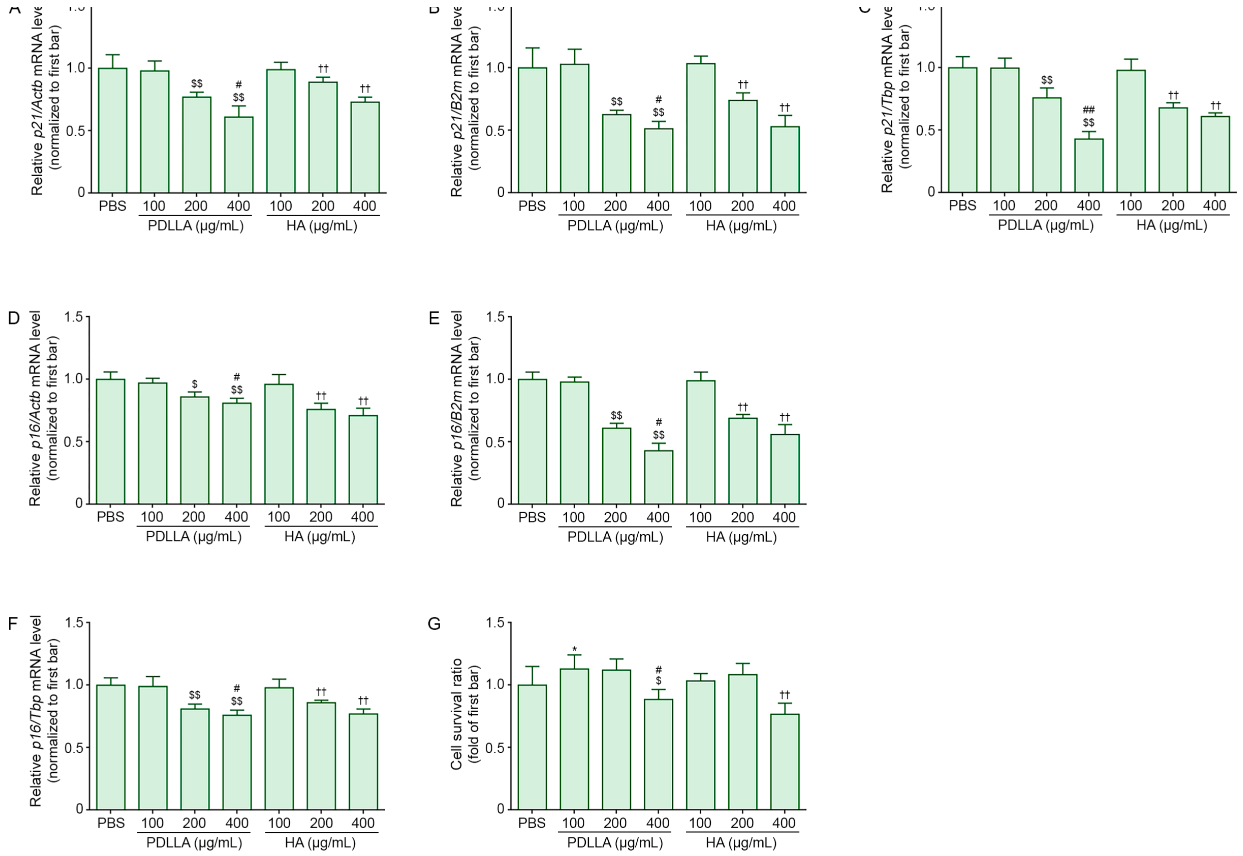

Figure S3. The optimal concentration of PDLLA or HA to reduce senescence in senescent macrophages. (**A–F**) To obtain the optimal concentration of PDLLA or HA for the treatment of senescent macrophages (RAW 264.7), the mRNA expression of p21 and p16 (senescence markers) was measured by qRT-PCR. The mRNA expression of p21 and p16 was unchanged at 100 µg/mL PDLLA or HA and decreased at 200 µg/mL. Data were normalized to Actb, B2m, or Tbp, and expression levels were reported according to the comparative CT method (ΔΔCT) relative to the first bar in the graph. (**G**) To determine the concentration at which treatment with PDLLA or HA did not result in cell death, cell survival assays were performed. Cell death did not occur at 100 and 200 µg/mL of PDLLA or HA, but it did occur at 400 µg/mL of PDLLA or HA. The data was analyzed relative to the mean of the first bar of the graph. Data are presented as the mean ± SD (n = 3/group). *, $p < 0.05$, first bar vs. second bar; $ or $$, $p < 0.05$ or $p < 0.01$, second bar vs. third bar or fourth bar; # or ##, $p < 0.05$ or $p < 0.01$, third bar vs. fourth bar; ††, $p < 0.01$, fifth bar vs. sixth bar or seventh bar.

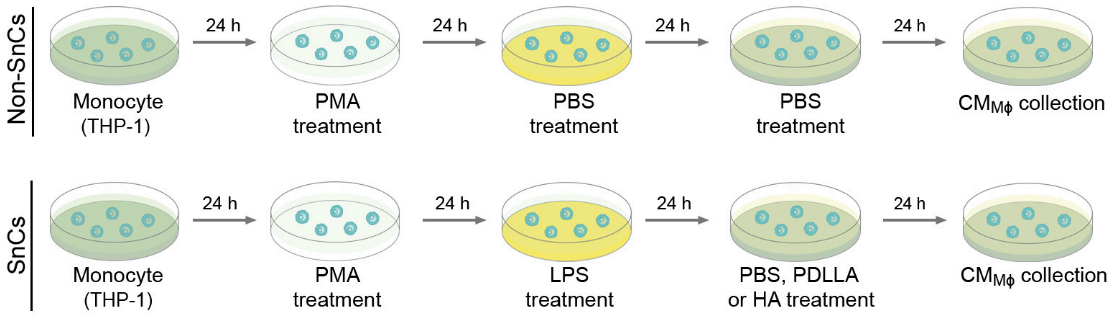

Figure S4. Schematic diagram to confirm the efficacy of PDLLA in senescent macrophages. This is an in vitro diagram to evaluate the efficacy of PDLLA in senescent macrophages. Human monocytes (THP-1) were treated with PMA (100 ng/mL) for 24 h to differentiate into macrophages. To induce senescence, macrophages were treated with LPS (1 μg/mL) for 24 h, followed by PDLLA or HA (200 μg/mL) for 24 h. Then, supernatant (conditioned medium from human macrophages; $CM_{M\Phi}$) was collected for co-culture with ASCs or fibroblasts.

.

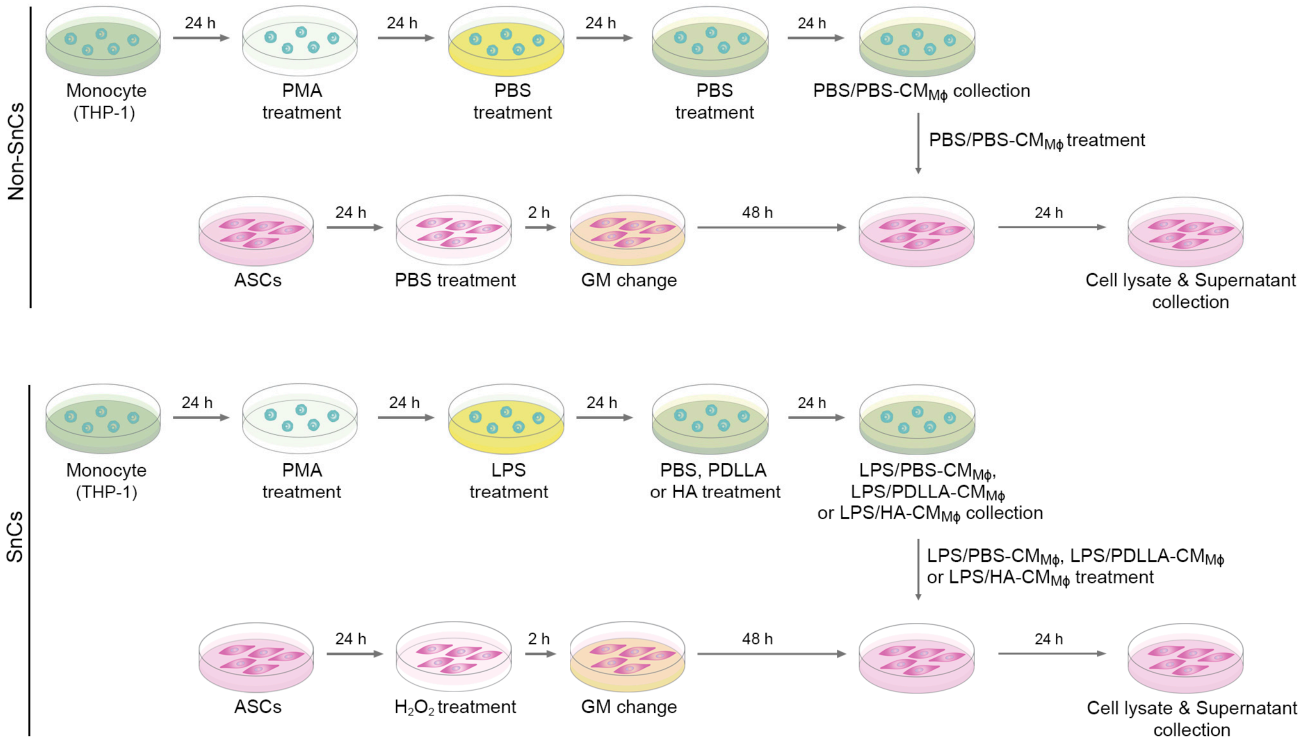

Figure S5. Schematic diagram to confirm the regulation of senescent ASCs proliferation and paracrine secretion by PDLLA-treated senescent macrophages. This is an *in vitro* diagram to evaluate the efficacy of PDLLA in senescent ASCs by modulating macrophages. To induce senescence, ASCs were treated with H_2O_2 (200 µM) for 2 h and then cultured in GM for 48 h. The ASCs culture medium was then changed to a mixture (1:1 ratio) of GM and $CM_{MΦ}$ treated with LPS (LPS/PBS-$CM_{MΦ}$), PDLLA (LPS/PDLLA-$CM_{MΦ}$), or HA (LPS/HA-$CM_{MΦ}$), respectively. The ASCs were then cultured for another 24 h, and cell lysates and supernatants (CM_{ASCs}) were collected.

.

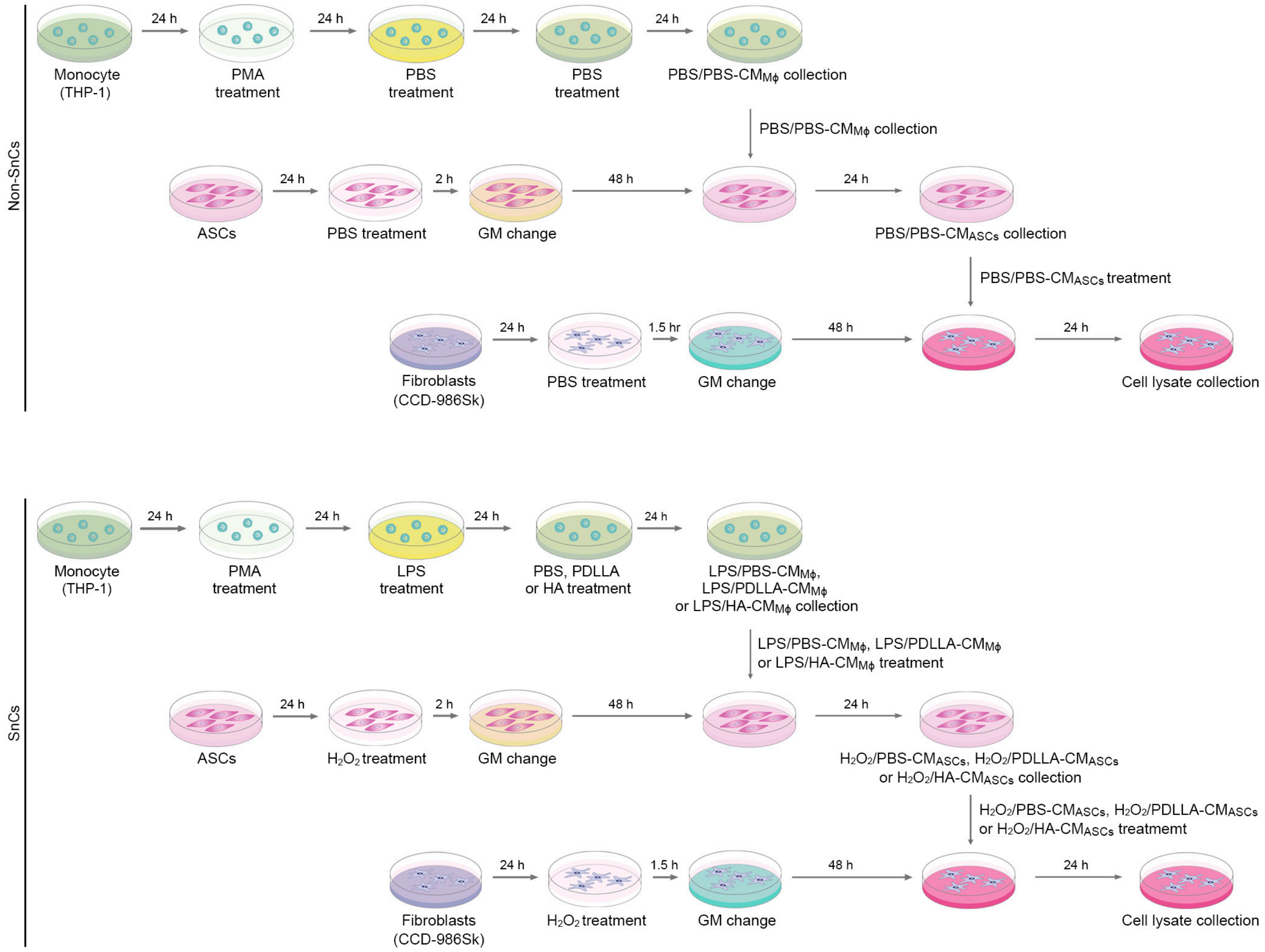

Figure S6. Schematic diagram to confirm the modulation of senescent fibroblast proliferation and collagen synthesis by PDLLA-$CM_{M\Phi}$-treated senescent ASCs. This is an in vitro diagram to evaluate whether the modulation of ASCs function by CM from PDLLA-treated macrophages affects fibroblast senescence. Fibroblasts (CCD-986Sk) were treated with H_2O_2 (350 µM) to induce senescence and then with a mixture (1:1 ratio) of GM and CM from ASCs that had been treated with CM from macrophages with or without HA or PDLLA treatment. Cell lysates of the fibroblasts were then collected.

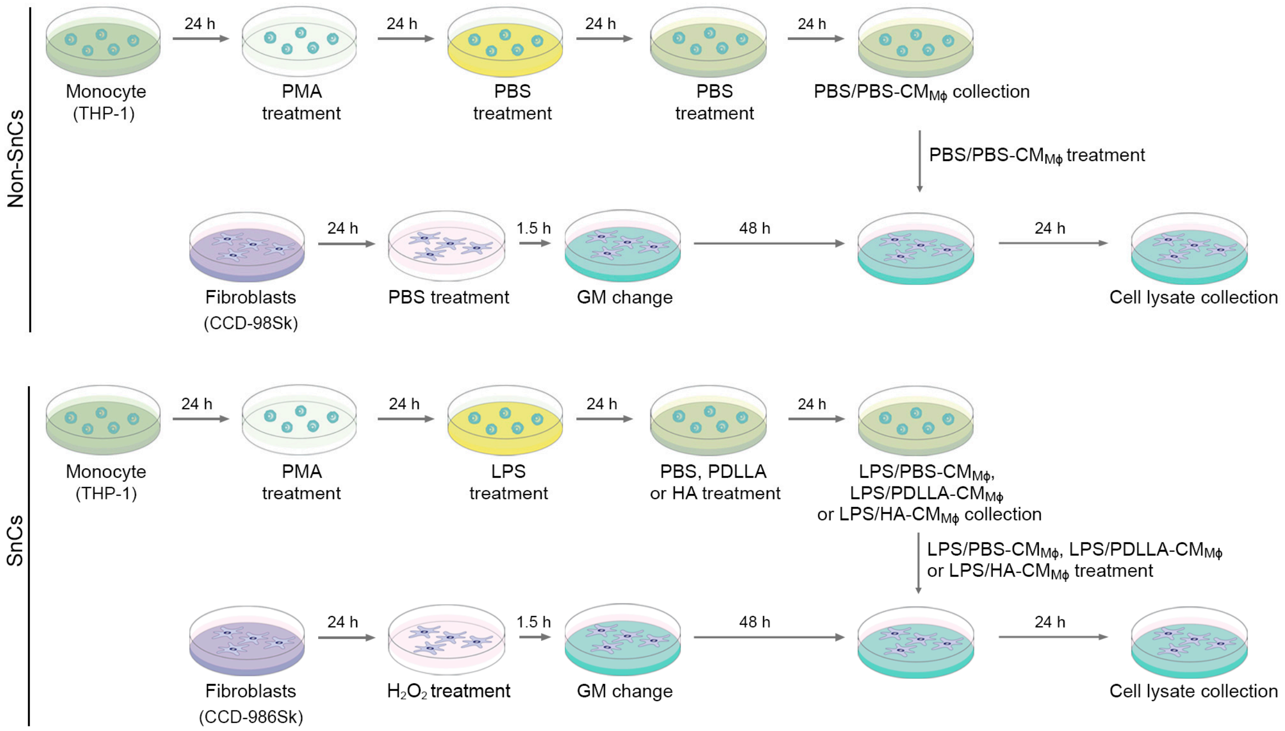

Figure S7. Schematic diagram to confirm the modulation of senescent fibroblast proliferation and collagen synthesis by PDLLA-treated senescent macrophages. This is an *in vitro* diagram to evaluate the modulation of fibroblast function by CM from PDLLA-treated macrophages. Fibroblasts (CCD-986Sk) were treated with H_2O_2 to induce senescence and then with a mixture (1:1 ratio) of GM and CM from macrophages with or without HA or PDLLA treatment. Cell lysates of the fibroblasts were then collected.

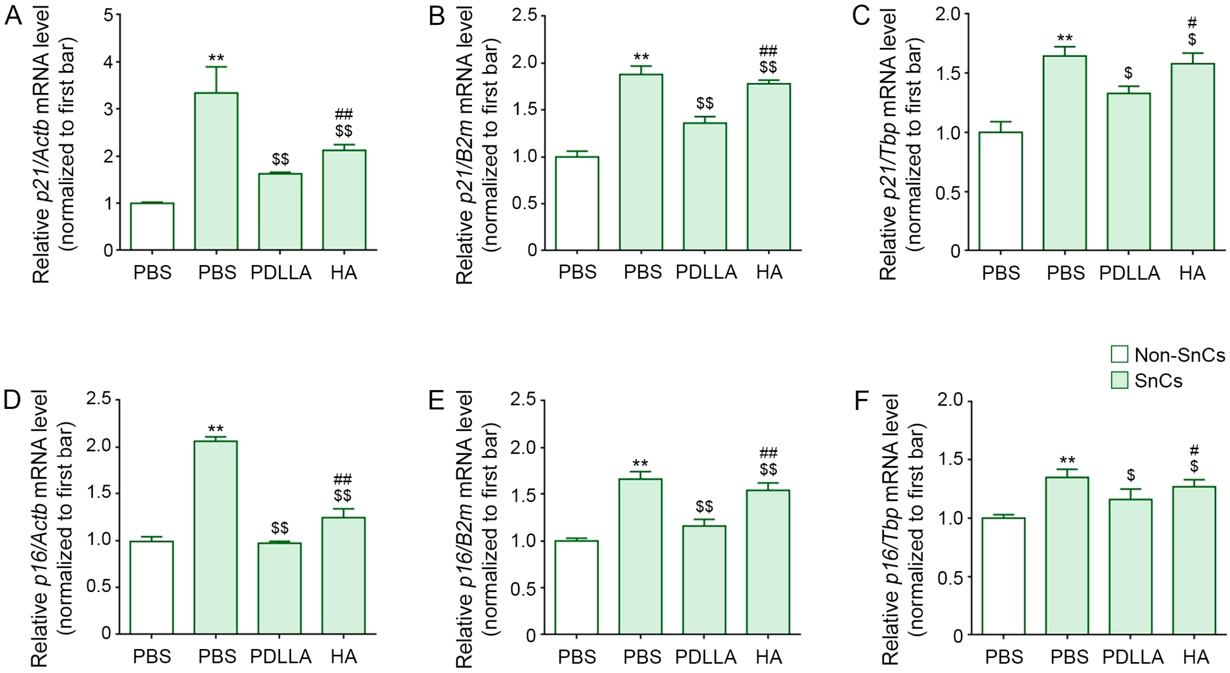

Figure S8. The reduction effect of senescence in H_2O_2-induced senescence murine macrophages by PDLLA treatment. (**A–F**) The mRNA expression levels in H_2O_2-induced senescent macrophages (RAW 264.7 cell) were validated using qRT-PCR. The mRNA expression of p21 and p16 (senescence markers) was increased by H_2O_2/PBS and reduced by H_2O_2/PDLLA or H_2O_2/HA (senescent cells, SnCs) compared with that in PBS/PBS (non-senescent cells, Non-SnCs). Data were normalized to Actb, B2m, or Tbp. Expression levels were reported according to the comparative CT method (ΔΔCT) relative to the first bar of the graph. Data are presented as the mean ± SD ($n = 3$/group). **, $p < 0.001$, first bar vs. second bar; $ and $$, $p < 0.05$ and $p < 0.01$, second bar vs. third bar or fourth bar; # and ##, $p < 0.05$ and $p < 0.01$, third bar vs. fourth bar.

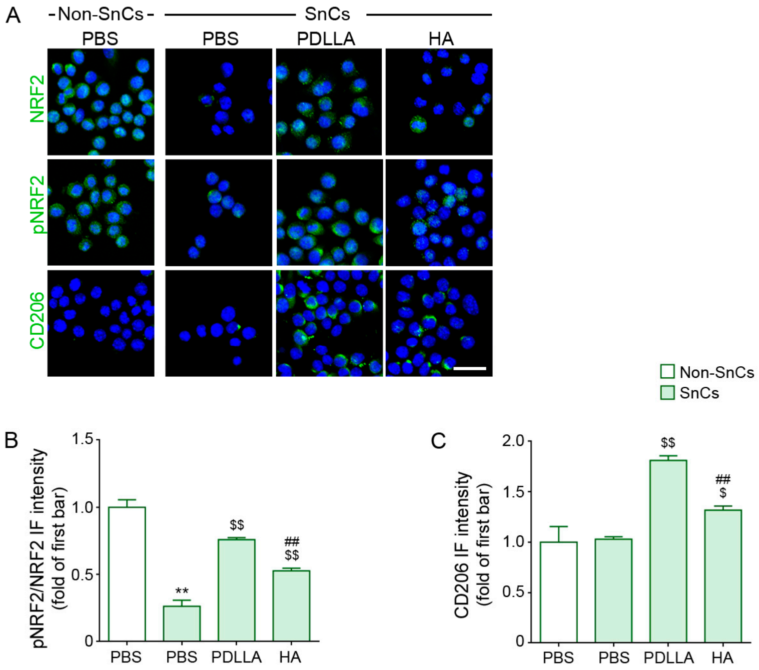

Figure S9. Upregulated pNRF2 activation and M2 polarization by PDLLA or HA treatment in senescent macrophages. (**A**) The expression levels of NRF2, pNRF2, and CD206 (M2 marker) in H_2O_2-induced senescent macrophages (RAW 264.7) were validated using immunocytochemistry (green: positive signals; scale bar = 20 µm). (**B**) This graph quantifies the data in the first and second lanes of Figure S5A. The expression of NRF2/pNRF2 was decreased by H_2O_2 treatment (senescent cells, SnCs) and was increased by PDLLA or HA treatment compared with non-senescent cells (Non-SnCs). (**C**) This graph quantifies the data in the third lane of Figure S5A. The expression of CD206 in H_2O_2-treated macrophages (SnCs) was unchanged by PBS treatment (Non-SnCs) and increased by PDLLA or HA treatment. The data was represented relative to the mean of the first bar of the graph. Data are presented as the mean ± SD (n = 3/group). **, $p < 0.01$, first bar vs. second bar; $ and $$, $p < 0.05$ and $p < 0.01$, second bar vs. third or fourth bar; ##, $p < 0.01$, third bar vs. fourth bar.

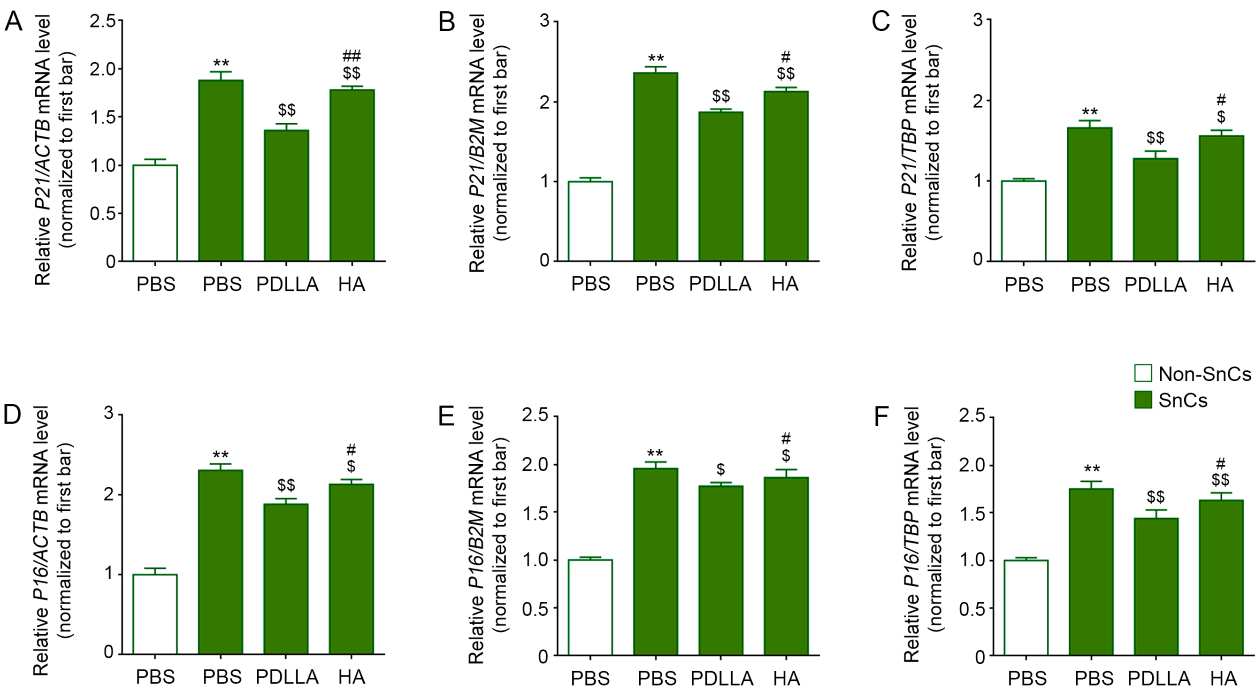

Figure S10. The reduction effect of senescence in senescent macrophages by PDLLA treatment. (**A–F**) The mRNA expression levels in LPS-induced senescent human macrophages (THP-1) were validated using qRT-PCR. The mRNA expression of p21 and p16 (senescence markers) was increased by LPS/PBS and reduced by LPS/PDLLA or LPS/HA (senescent cells, SnCs) compared with that in PBS/PBS (non-senescent cells, Non-SnCs). Data were normalized to ACTB, B2M, or TBP. Expression levels were reported according to the comparative CT method (ΔΔCT) relative to the first bar in the graph. Data are presented as the mean ± SD (n = 3/group). **, $p < 0.001$, first bar vs. second bar; $ and $$, $p < 0.05$ and $p < 0.01$, second bar vs. third bar or fourth bar; # and ##, $p < 0.05$ and $p < 0.01$, third bar vs. fourth bar.

.

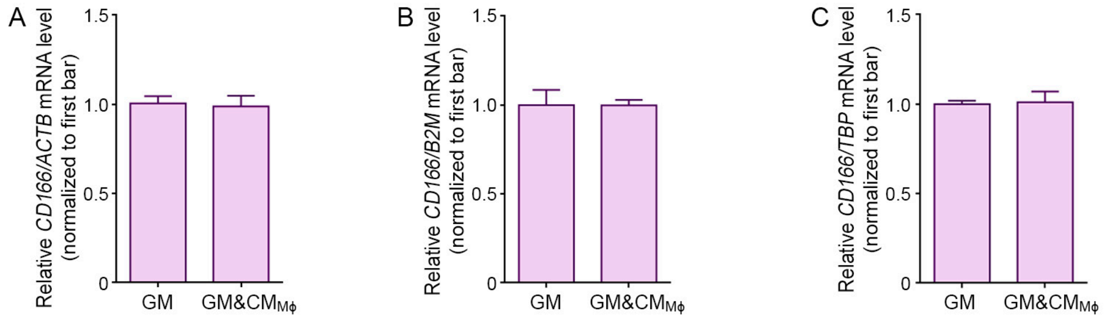

Figure S11. Changes in the character of ASCs upon treatment with a mixture of CM$_{M\Phi}$ and growth medium. (**A–C**) To assess whether the cell culture mixture could alter ASCs properties, mRNA expression levels in ASCs were determined by qRT-PCR. The mRNA expression of CD166 (ASCs marker) in ASCs was unchanged by the mixture of CM$_{M\Phi}$ and growth medium. Data were normalized to *ACTB*, *B2M*, or *TBP*. Expression levels were reported according to the comparative CT method (ΔΔCT) relative to the first bar in the graph. Data are presented as the mean ± SD (*n* = 3/group).

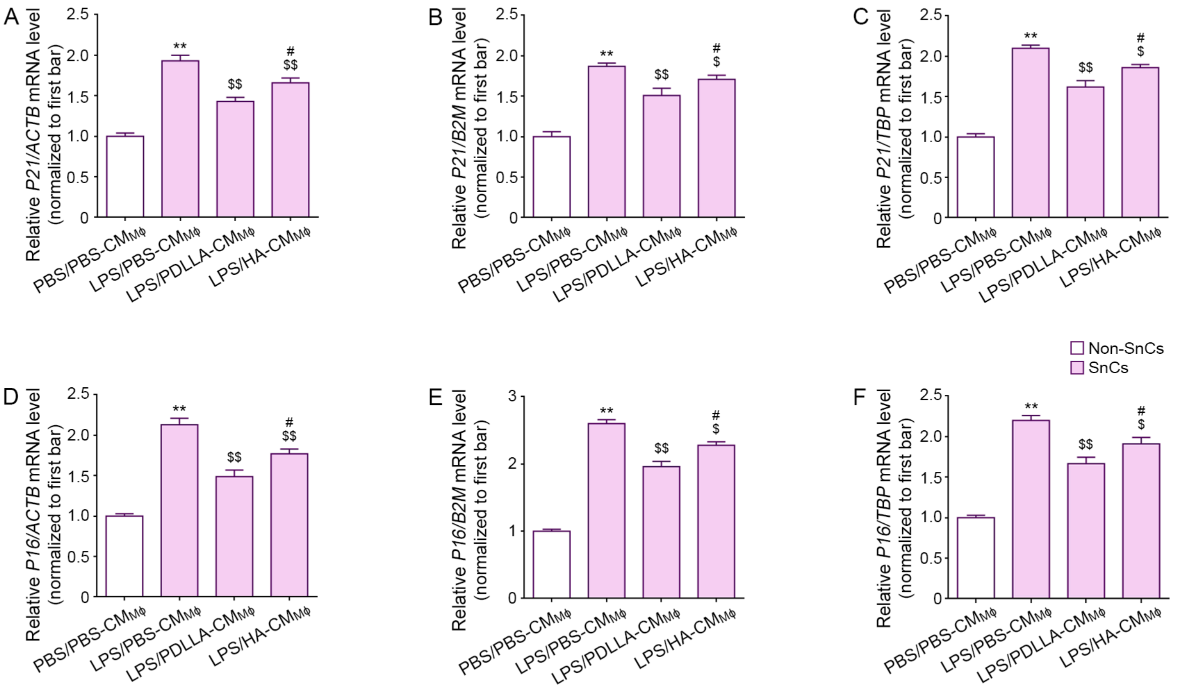

Figure S12. Reduced effect of senescence on ASCs via modulation of macrophages treated with PDLLA. (**A–F**) The mRNA expression levels in H_2O_2-induced senescent ASCs were determined by qRT-PCR. The mRNA expression of p21 and p16 (senescence markers) was increased in senescent ASCs treated with LPS/PBS-$CM_{M\Phi}$ and reduced in senescent ASCs (SnCs) treated with LPS/PDLLA-$CM_{M\Phi}$ or LPS/HA-$CM_{M\Phi}$ compared with that in non-senescent ASCs (Non-SnCs) treated with PBS/PBS-$CM_{M\Phi}$. Data were normalized to *ACTB, B2M*, or *TBP*. Expression levels were reported according to the comparative CT method (ΔΔCT) relative to the first bar in the graph. Data are presented as the mean ± SD (n = 3/group). **, $p < 0.01$, first bar vs. second bar; $ and $$, $p < 0.05$ and $p < 0.01$, second bar vs. third or fourth bar; #, $p < 0.05$, third bar vs. fourth bar.

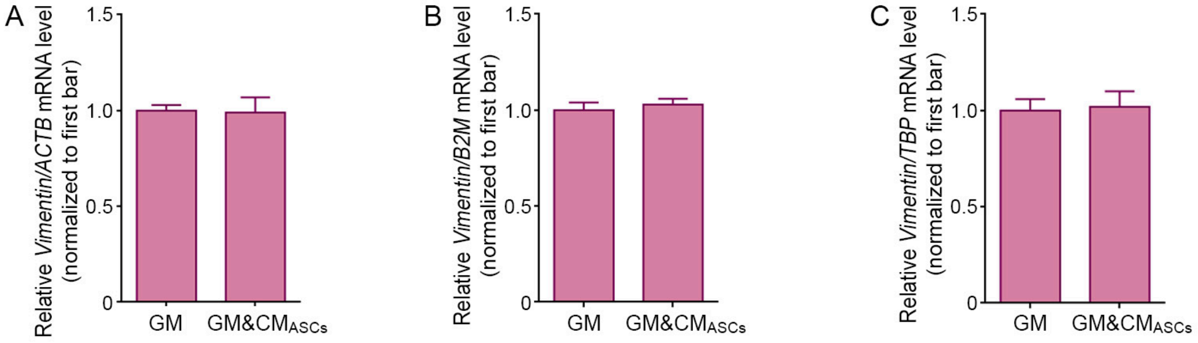

Figure S13. Changes in the character of fibroblasts upon treatment with CM$_{ASCs}$ and growth medium mixture. (**A–C**) To assess whether the cell culture mixture could alter the properties of fibroblasts, we measured mRNA expression levels in fibroblasts using qRT-PCR. The mRNA expression of vimentin (a fibroblast marker) was unchanged by the mixture of CM$_{ASCs}$ and growth medium compared to growth medium alone. Data were normalized to ACTB, B2M, or TBP. Expression levels were reported according to the comparative CT method (ΔΔCT) relative to the first bar in the graph. Data are presented as the mean ± SD (n = 3/group).

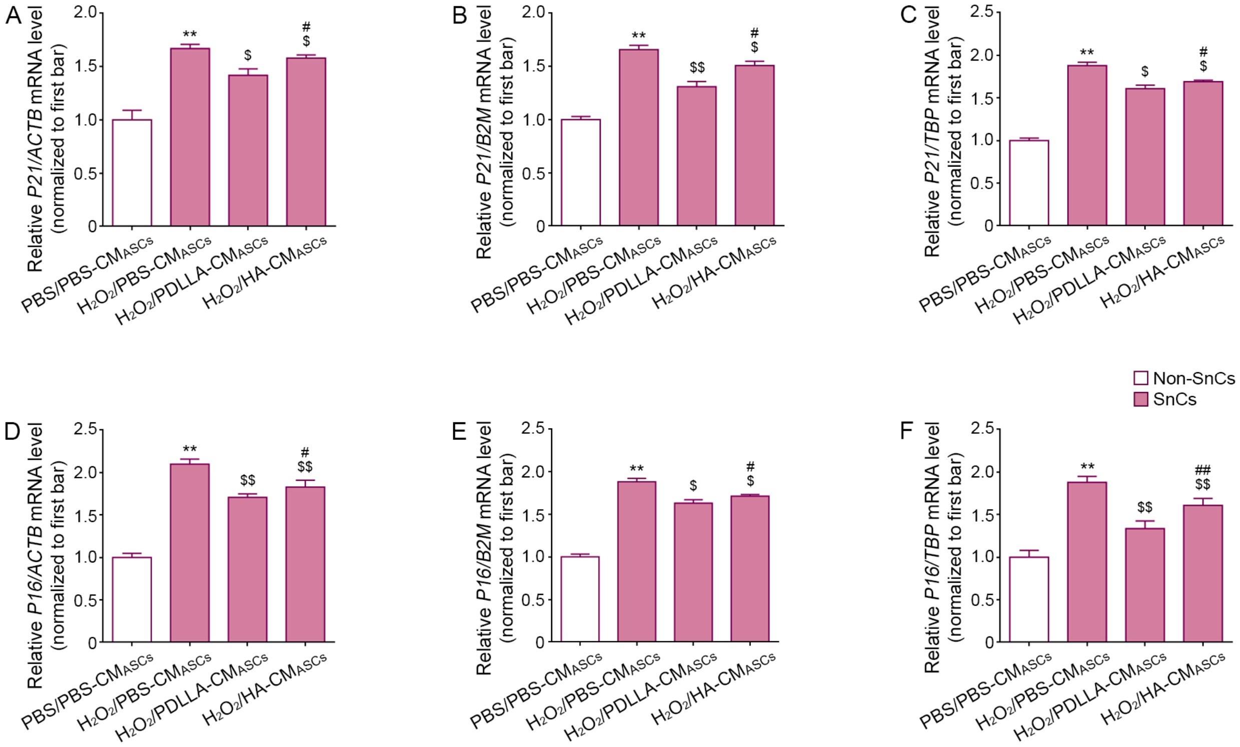

Figure S14. Reduced effect of senescence on fibroblast via modulation of ASCs affected with PDLLA. PDLLA modulation of ASCs reduced aging effects in H_2O_2-induced senescent fibroblasts. (A–F) The mRNA expression levels in H_2O_2-induced senescent fibroblasts were measured by qRT-PCR. The mRNA expression of p21 and p16 (senescence markers) was increased by H_2O_2/PBS-CM_{ASCs} and reduced by H_2O_2/PDLLA-CM_{ASCs} or H_2O_2/HA-CM_{ASCs} in senescent cells (SnCs) compared with that in non-senescent ASCs treated with PBS/PBS-$CM_{M\Phi}$ (Non-SnCs). Data were normalized to *ACTB*, *B2M*, or TBP. Expression levels were reported according to the comparative CT method (ΔΔCT) relative to the first bar in the graph. Data are presented as the mean ± SD (n = 3/group). **, $p < 0.01$, first bar vs. second bar; $ and $$, $p < 0.05$ and $p < 0.01$, second bar vs. third or fourth bar; # and ##, $p < 0.05$ and $p < 0.01$, third bar vs. fourth bar.

.

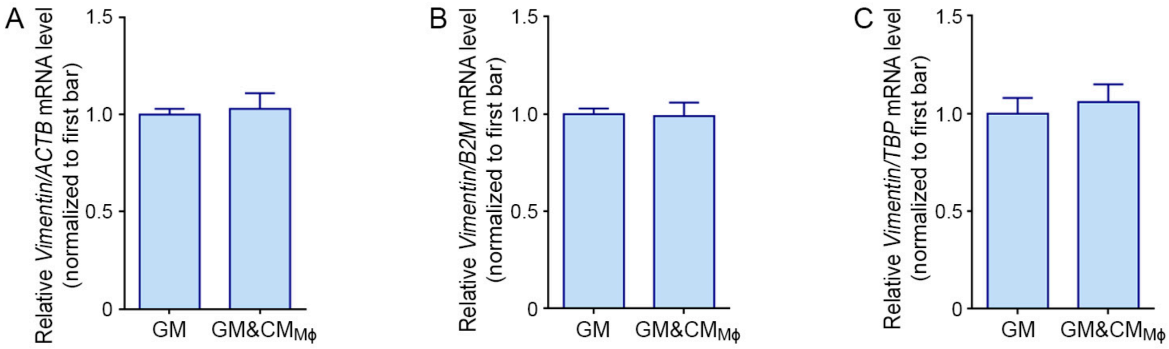

Figure S15. The $CM_{MΦ}$ and growth medium mixture did not change the character of fibroblasts. (**A–C**) To assess whether the cell culture mixture could alter fibroblast properties, we measured vimentin (fibroblast marker) mRNA levels by qRT-PCR. The mRNA expression of vimentin in fibroblasts was unchanged by the $CM_{MΦ}$ and growth medium treatment compared with growth medium treatment alone. Data were normalized to ACTB, B2M, or TBP. Expression levels were reported according to the comparative CT method (ΔΔCT) relative to the first bar in the graph. Data are presented as the mean ± SD (*n* = 3/group).

.

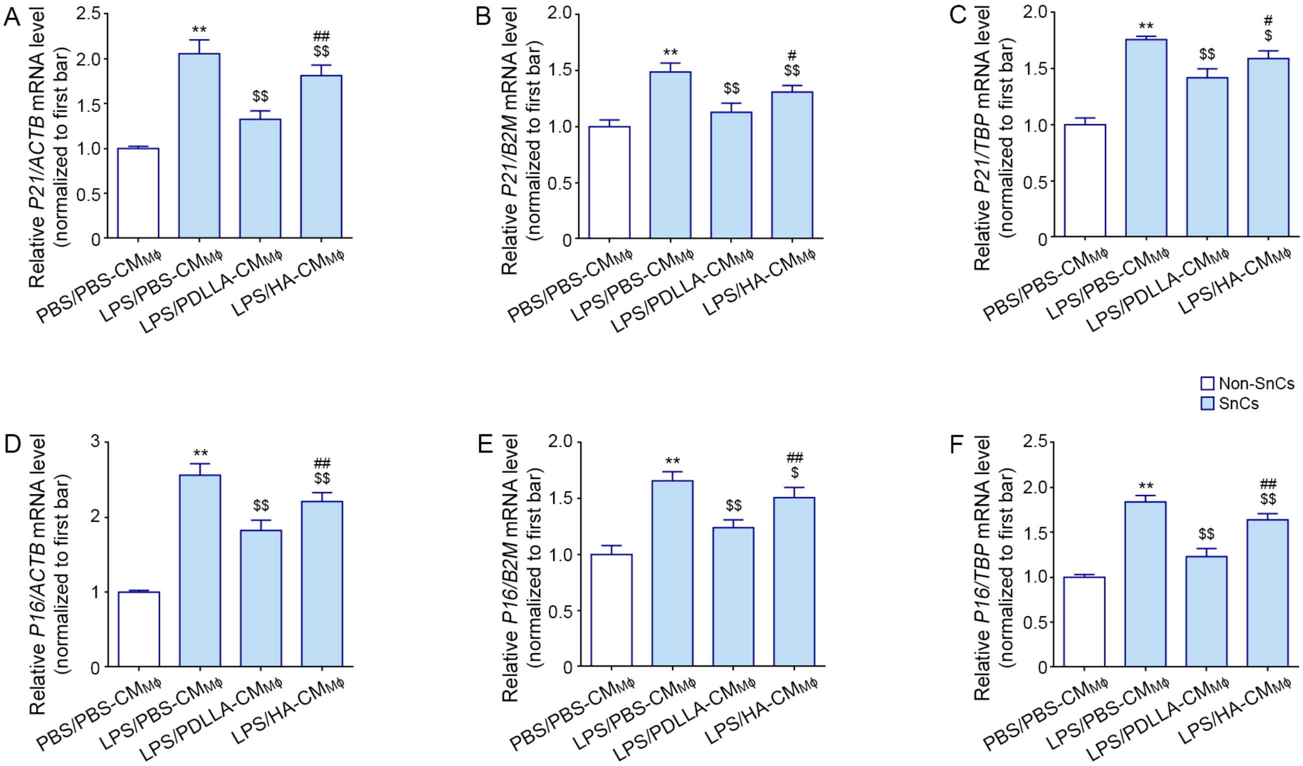

Figure S16. Reduced effect of senescence on fibroblasts via modulation of macrophages treated with PDLLA. (**A–F**) The mRNA expression levels in H_2O_2-induced senescent fibroblasts were measured using qRT-PCR. The mRNA expression of p21 and p16 (senescence markers) was increased in senescent cells (SnCs) cultured with LPS/PBS-$CM_{MΦ}$ compared with that in non-senescent cells (Non-SnCs) treated with PBS/PBS-$CM_{MΦ}$. SnCs cultured with LPS/PBS-$CM_{MΦ}$ had higher p21 and p16 expression than SnCs cultured with LPS/PDLLA-$CM_{MΦ}$ or LPS/HA-$CM_{MΦ}$. Data were normalized to ACTB, B2M, or TBP. Expression levels were reported according to the comparative CT method (ΔΔCT) relative to the first bar in the graph. Data are presented as the mean ± SD (n = 3/group). **, $p < 0.01$, first bar vs. second bar; $ and $$, $p < 0.05$ and $p < 0.01$, second bar vs. third or fourth bar; # and ##, $p < 0.05$ and $p < 0.01$, third bar vs. fourth bar.

.

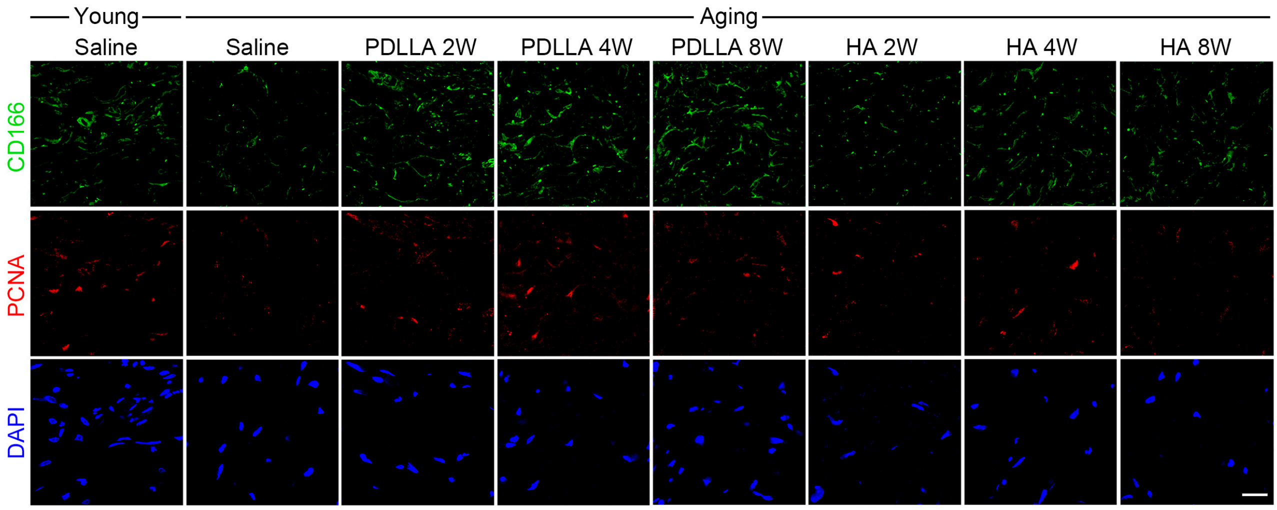

Figure S17. Upregulatory effects of PDLLA on ASCs proliferation in aged skin. The co-stained fluorescence levels of CD166 (ASCs marker; green) and PCNA (proliferation cell marker; red) were validated by immunofluorescence (nuclei; blue) (scale bar = 50 μm).

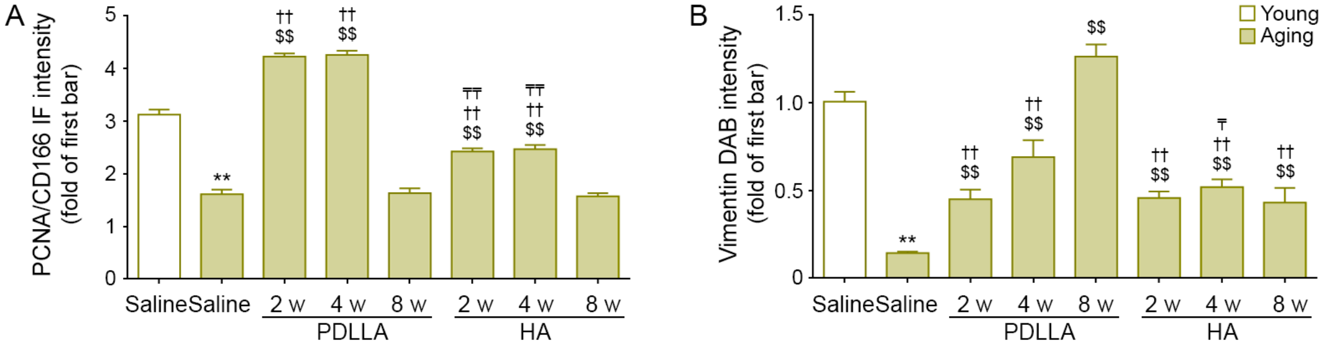

Figure S18. Upregulatory effects of PDLLA on dermal fibroblast proliferation in aged skin. (**A**) This graph quantifies the data in Figure 4A. The proliferation of ASCs was reduced in saline-injected aged skin compared with that in saline-injected young skin and was increased in aged skin by PDLLA or HA, except at 8 weeks after injection. (**B**) This graph quantifies the data in Figure 4B. The intensity of vimentin was reduced in aged skin compared with that in young skin and was increased in aged skin by PDLLA or HA treatment. The data was represented relative to the mean of the first bar of the graph. Data are presented as the mean ± SD (n = 3/group). **, $p < 0.001$, first bar vs. second bar; $$, $p < 0.01$, second bar vs. third bar~eighth bar; ††, $p < 0.01$, fifth bar vs. third bar~eighth bar; ͳ and ͳͳ, $p < 0.05$ and $p < 0.01$, eighth bars vs. sixth bar or seventh bar.

.

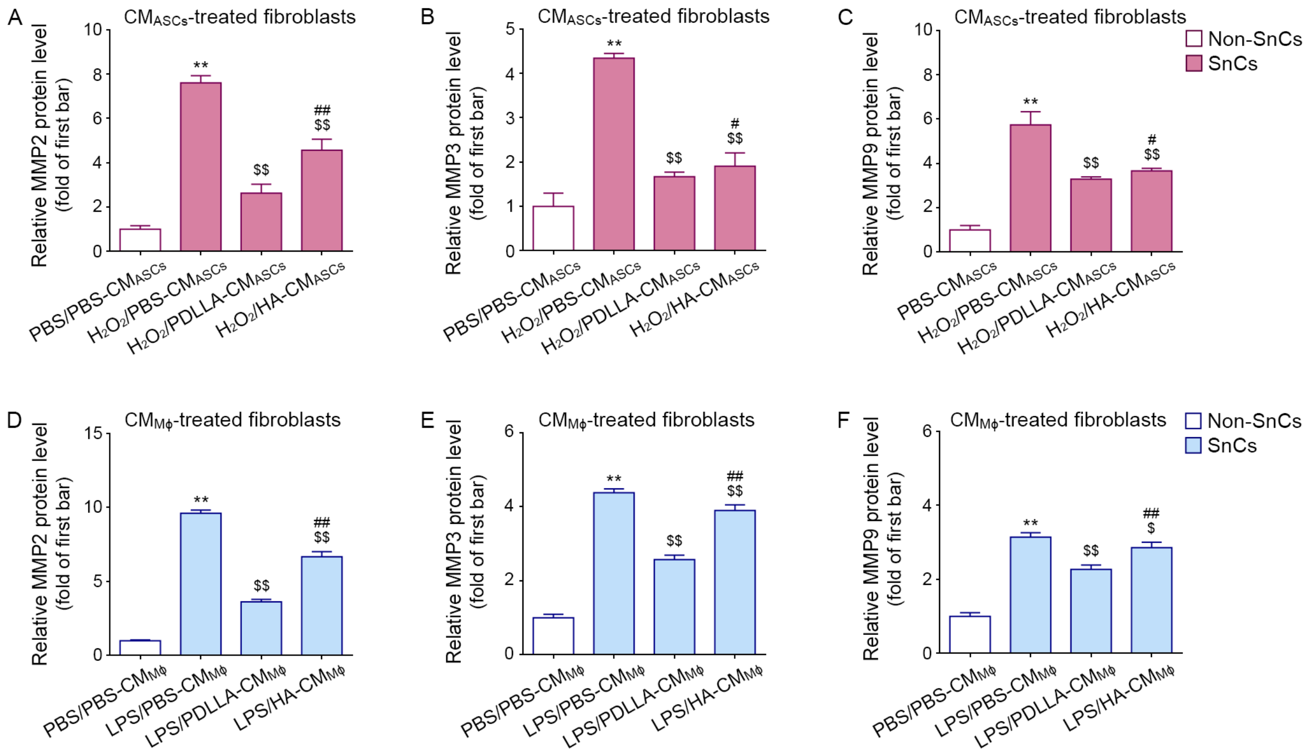

Figure S19. Reduced MMPs expression in senescent fibroblasts by PDLLA via effects on macrophages and ASCs. (**A–C**) These graphs quantify the data in Figure 5C. The expression of MMP2, MMP3, and MMP9 was increased by H_2O_2/H_2O_2-CM$_{ASCs}$ compared with that in control fibroblasts. The increased expression was attenuated in fibroblasts treated with H_2O_2/PDLLA-CM$_{ASCs}$ or H_2O_2/HA-CM$_{ASCs}$. (**D–F**) This graph quantifies the data in Figure 5D. The expression of MMP2, MMP3, and MMP9 was increased in fibroblasts treated with LPS/H_2O_2-CM$_{M\Phi}$ compared with that in control fibroblasts. The increase in MMP2, MMP3, and MMP9 expression was attenuated in fibroblasts treated with LPS/PDLLA-CM$_{M\Phi}$ or LPS/HA-CM$_{M\Phi}$. To correct for differences in protein loading, the quantification of the western blot was normalized using β-actin as a loading control protein. For each blot, the values were expressed relative to the mean of the first bar in the graph. Data are presented as the mean ± SD (n = 3/group). **, $p < 0.01$, first bar vs. second bar; $ and $$, $p < 0.05$ and $p < 0.01$, second bar vs. third bar or fourth bar; # and ##, $p < 0.05$ and $p < 0.01$, third bar vs. fourth bar.

.

Table S1. List of primer for qRT-PCR in this study.

Gene	Host	Primers	
Actb	Mouse	Forward	5'-AGAAGGACTCCTATGTGGGTGA-3′
		Reverse	5'-GGCATAGAGGTCTTTACGGATG-3′
B2m		Forward	5'-TTCAAGTATACTCACGCCACCC-3′
		Reverse	5'-GCAGGCGTATGTATCAGTCTCA-3′
Tbp		Forward	5'-TCCACAGCCTATTCAGAACACC-3′
		Reverse	5'-TGAGAATGGAAGAGTTGTGGGG-3′
p21		Forward	5′-GAGAACGGTGGAACTTTGACTT-3′
		Reverse	5′-CTCAGACACCAGAGTGCAAGAC-3′
p16		Forward	5′-CAACGCACCGAATAGTTACG-3′
		Reverse	5′-ATCTATGCGGGCATGGTTACT-3′
ACTB	Human	Forward	5′-CCACGAAACTACCTTCAACTCC-3′
		Reverse	5′-ACTCGTCATACTCCTGCTTGCT-3′
B2M		Forward	5'-TGGGTTTCATCCATCCGACATT-3′
		Reverse	5'-AACCTCCATGATGCTGCTTACA-3′
TBP		Forward	5'-GGAGAGTTCTGGGATTGTACCG-3′
		Reverse	5'-CTGTTCTTCACTCTTGGCTCCT-3′
P21		Forward	5′-ATGTGGACCTGTCACTGTCTTG-3′
		Reverse	5′-CTTCCTCTTGGAGAAGATCAGC-3′
P16		Forward	5′-GCTCAACTACGGTGCAGATTC-3′
		Reverse	5′-AATATCGCACGATGTCTTGATG-3′
CD166		Forward	5'-AAGGAAATGGACCCAGTGACTC-3′
		Reverse	5'-TTTGGTGGCAGCACTTGTATTG-3′
Vimentin		Forward	5'-TCAGAGAGAGGAAGCCGAAAAC-3′
		Reverse	5'- CGATTTGGACATGCTGTTCCTG-3′

Table S2. List of antibodies used in this study.

Antibody	Company	Catalog no.	Dilution rate				
			ICC	IF	DAB	ELISA	WB
NRF2	Bioss	bs-1074R	1:200		1:200		
pNRF2	Bioss	bs-2013R	1:200		1:200		
CD206	Novus	NBP1-90020	1:100		1:200		
IL-10	FineTest	FNab04211				1:1000:	
TGF-β	Abcam	Ab64715				1:1000	
FGF2	Abcam	Ab106245				1:1000	
CD166	HUABIO	M1012-7		1:100			
PCNA	Abcam	Ab18197		1:50			
COL1A1	Santa Cruz Biotechnology	sc-293182				1:100	
COL3A1	Bioss	Bs-0549R				1:1000	
EBP	LSBio	LS-C167605-400				1:1000	
ELN	MyBioSource	MBS821275				1:1000	
Vimentin	Santa Cruz Biotechnology	sc-373717			1:200		
NF-κB	Cell signaling	8242s	1:200		1:200		
MMP2	LSBIO	LS-C352523					1:500
MMP3	CUSABIO	CSB-PA17509A0Rb					1:2000
MMP9	GeneTEX	GTX31891					1:1000
β-actin	Cell signaling	4967					1:1000
